# A Quality Analysis of the Measurement Properties of the Clinician-Reported Outcome Measures for Vitiligo and of the Studies Assessing Them: A Systematic Review

**DOI:** 10.3390/jcm14082548

**Published:** 2025-04-08

**Authors:** Jolien Duponselle, Sandrine Herbelet, Liesbeth Delbaere, Zoë De Schryver, Maxine Forman, Caroline B. Terwee, Albert Wolkerstorfer, Julien Seneschal, Phyllis I. Spuls, Amit Garg, Iltefat Hamzavi, Reinhart Speeckaert, Nanja van Geel

**Affiliations:** 1 Department of Dermatology, Ghent University Hospital, 9000 Ghent, Belgium; 2 Department of Epidemiology and Data Science, Amsterdam University Medical Centers, Vrije Universiteit Amsterdam, 1081 BT Amsterdam, The Netherlands; 3 Amsterdam Public Health Research Institute, Methodology, 1105 AZ Amsterdam, The Netherlands; 4 Department of Dermatology, Academic Medical Center, 1105 AZ Amsterdam, The Netherlands; 5 INSERM U1035, Biotherapy of Genetic Diseases, Inflammatory Disorders and Cancers (BMGIC), Immunodermatology ATIP-AVENIR, University of Bordeaux, FHU ACRONIM, 33076 Bordeaux, France; 6 Department of Dermatology and Pediatric Dermatology, National Reference Center for Rare Skin Disorders, Hôpital Saint-André, 33076 Bordeaux, France; 7 Department of Dermatology, Northwell Health, New York, NY 10028, USA; 8 Department of Dermatology, Henry Ford Hospital, Detroit, MI 48202, USA

**Keywords:** clinician-reported outcome measures, measurement properties, vitiligo, COSMIN guidelines

## Abstract

**Background/Objective:** Evaluating the measurement properties (MPs) of Clinician-Reported Outcome Measures (ClinROMs) is crucial for selecting appropriate instruments for vitiligo assessment. This review critically appraises the existing evidence on the MPs of the ClinROMs used in vitiligo. **Methods:** A systematic search was conducted in PubMed, Embase, and the Cochrane Library up to 20 February 2024, identifying validated ClinROMs in vitiligo. Studies were included if they provided original data on ClinROM development or analysis, excluding those solely validating other instruments. The assessment of ClinROM quality and risk of bias analysis followed COSMIN guidelines, and ClinROMs with the highest number of sufficiently rated MPs supported by a moderate/high Quality of Evidence (QoE) were identified per construct category (extent/repigmentation and evolution/activity). **Results:** This review included 22 studies evaluating 12 ClinROMs. For extent/repigmentation, the Vitiligo Area and Severity Index (VASI), Vitiligo Extent Score (VES), and VESplus each had four MPs rated sufficient with a moderate/high QoE. For evolution, the Vitiligo Disease Improvement Score (VDIS) and Vitiligo Disease Activity Score (VDAS) similarly had four MPs rated sufficient with a moderate/high QoE. For activity evaluated based on a single time point, the Vitiligo Signs of Activity Score (VSAS), the only validated ClinROM for visible signs of disease activity, had three MPs rated sufficient with a moderate/high QoE. **Conclusions:** Six ClinROMs demonstrated the highest quality ratings across two key constructs. However, none underwent a complete evaluation of all their MPs, highlighting the need for further validation and refinement.

## 1. Introduction

Vitiligo, affecting 0.1–1% of the global population, results in white patches due to melanocyte loss. Its impact is psychologically significant [1].

To study and effectively manage this condition, the utilization of Clinician-Reported Outcome Measures (ClinROMs) has emerged as a crucial strategy across diverse healthcare settings [2]. These instruments measure specific facets of vitiligo, including disease extent/repigmentation and disease activity/evolution. The selection of high-quality measurement instruments requires a comprehensive understanding of various quality aspects, collectively referred to as measurement properties (MPs). These MPs include the following [3,4,5]:

1. Content Validity: The degree to which the instrument is relevant, comprehensible, and comprehensive for its construct, target population, and context of use.
2. Structural Validity: The extent to which the instrument’s scores accurately reflect the dimensionality of the measured construct.
3. Internal Consistency: The degree of interrelatedness among items within a unidimensional scale or subscale.
4. Reliability: The consistency of scores in stable patients when measured under identical conditions.
5. Measurement Error: The amount of error in scores that is not due to true changes in the construct being measured.
6. Criterion Validity: The extent to which the instrument’s scores correspond to those of a gold-standard measure.
7. Construct Validity: The degree to which the scores align with predefined hypotheses based on the instrument’s theoretical framework.
8. Responsiveness: The capability of the instrument to detect meaningful changes in the construct over time.
9. Cross-Cultural Validity: The ability of a translated or adapted instrument to maintain comparable performance to the original version.

Further details on MPs are available in Supplementary Material S3.

Currently, the existing literature lacks an up-to-date and comprehensive systematic review focusing on the analysis of MPs for the ClinROMs in vitiligo.

The analysis of MPs can be performed in accordance with the COnsensus-based Standards for the selection of health Measurement INstruments (COSMIN) framework [3,4,5]. COSMIN analysis involves an international multidisciplinary research team with expertise in the development/evaluation of outcome measurement instruments.

The quality assessment of vitiligo ClinROMs is part of the ongoing international vitiligo consensus project ‘Vitiligo International Task Force for an Agreed List of Core Data’ (VITAL). VITAL’s primary mission is to establish global standardization for the assessment/monitoring of vitiligo. Employing a consensus-driven methodology, VITAL seeks to offer recommendations regarding the utilization of outcome measurement instruments in vitiligo across diverse settings (registries, clinical practice, and clinical trials) [6]. The VITAL project is part of the broader Vitiligo Outcome Instruments and Consensus for Evidence (VOICE) initiative, operating under the umbrella of the CHORD-COUSIN Collaboration (C3). C3’s objective is to establish a uniform methodology for creating core outcome sets within the field of dermatology. The utilization of ClinROMs in dermatology represents a significant advancement, enabling the standardized and more objective evaluation of disease-specific outcomes. This not only enhances patient care but also ensures robust data collection for research and clinical trials, fostering evidence-based practices in the management of skin diseases such as vitiligo.

Within this context, this review’s primary aim was to provide an in-depth analysis of the available evidence concerning the MPs of ClinROMs in the context of vitiligo, also including the quality of the studies in which they were reported (risk of bias). The secondary aim was to identify the ClinROMs within each construct category (extent/repigmentation and evolution/activity) with the highest number of sufficiently rated MPs with a moderate/high Quality of Evidence.

## 2. Methods

### 2.1. Search Strategy and Selection Criteria

A search was conducted across three electronic databases—PubMed, Embase, and the Cochrane Library [CENTRAL]—as detailed in Supplementary Material S1. The search strategy was in accordance with the COSMIN standards and was developed by M.F., T.A. and A.V.B. in collaboration with the information specialist N.P. and methodology expert C.T. The search was designed to include ClinROMs and Patient-Reported Outcome Measures (PROMs)—relevant to a concurrent PROMs review (concurrently submitted with the current review)—both registered with PROSPERO (CRD42020216338). The key search terms used were as follows: “vitiligo”, “validation study”, and “outcome assessment”. A complete list of search terms is provided in Supplementary Material S1. This review was conducted according to the Preferred Reporting Items for Systematic Reviews and Meta-Analyses (PRISMA) statement.

The studies eligible for inclusion involved vitiligo patients (at least 25% or separate analysis) and reported original data related to ClinROM quality analysis or development. There was no restriction for age, sex, ethnicity, type of treatment, or type of vitiligo. ClinROMs are defined in this review as instruments used by a healthcare provider which report on a patient’s health status. If their primary objective was solely the validation of another tool, studies were not included. Further insights into the inclusion and exclusion criteria are elaborated in Supplementary Material S2.

The initial step in the selection process was the removal of duplicates using EndNote X9 (Clarivate Analytics, London, UK). Subsequent evaluations of the studies’ suitability were conducted using the Covidence software 2021 (Covidence systematic review software, Veritas Health Innovation, Melbourne, Australia, accessible at www.covidence.org). In the initial search, each record underwent screening (abstract/title and full text) by two independent reviewers (M.F. and T.A.). Three additional search updates were executed, the first update screening (abstract/title and full text) was performed by the reviewers L.D. and J.D., the second update and third update screening (abstract/title and full text) was performed by J.D. and in addition, a post-hoc, independent similar third update screening was performed by Z.D.S. (full-text).

### 2.2. Data Extraction and Quality Assessment

The characteristics of the ClinROMs and the study populations were systematically collected and cataloged by one reviewer (J.D.).

Following this initial phase, a quality analysis focusing on the MPs of ClinROMs was undertaken by three assessors (L.D., S.H., J.D.). Every reviewer conducted a full independent analysis, ensuring a profound and unbiased approach. A comprehensive outline of the MPs, along with their explicit definitions, is provided in Supplementary Material S3.

The analysis was conducted following the COSMIN guidelines. This involved assessing the risk of bias (RoB) in the included studies using the extensive COSMIN RoB checklist [3,4,5]. This checklist encompassed standards for design requirements and preferred statistical methods. Additionally, the study results were rated against the COSMIN criteria for good MPs. This process continued by summarizing the results from multiple studies focusing on the same MP of the same ClinROM and subsequently rating this consolidated result against the criteria for good MPs again. The approach culminated in an evaluation of the quality of the overall level of evidence for each MP of each ClinROM, employing a GRADE methodology. A detailed explanation of the analysis process can be found in Supplementary Material S4.

In the conclusive phase of the analysis, the advice of an expert review panel consisting of two vitiligo specialists (N.v.G., R.S.) on ambiguities that emerged during the analysis process was incorporated. Moreover, the methodology, emergent results, and remaining ambiguities were subjected to an in-depth review by COSMIN methodology expert C.T. Uncertainties related to statistics were referred to statistical expert R.C.

## 3. Results

An extensive search was conducted from inception until 16 February 2021, resulting in the retrieval of 3723 articles. After duplicate removal and the screening of titles and abstracts, 92 full-text articles were selected for detailed evaluation. Following the application of predefined inclusion criteria, 12 articles were deemed eligible for inclusion.

To ensure comprehensiveness, three search updates were performed on 13 July 2022, 26 June 2023, and 20 February 2024. These updates identified an additional eight articles that met the inclusion criteria. Furthermore, 2 additional articles were included through a manual search, bringing the total number of eligible studies to 22.

The complete search strategy is detailed in Supplementary Material S1. Additional agreements for supplementary analyses, established by the review team in accordance with the COSMIN guidelines, along with their rationale, are detailed in Supplementary Material S5. For instance, cross-cultural validity was excluded from the review as none of the included studies addressed it. Another example was the standard for a total sample size of at least 100, which is typically applied for PROMs, but is not in line with common practice in ClinROMs, as multiple raters are assessing the same, often smaller, patient population.

The characteristics of the included studies can be found in Supplementary Material S6.

The review included 12 ClinROMs:

1. Vitiligo Area and Severity Index (VASI).
2. Facial Vitiligo Area and Severity Index (F-VASI).
3. Vitiligo European Task Force assessment (VETFa).
4. Vitiligo Extent Score (VES).
5. Vitiligo Extent Score Plus (VESplus).
6. Vitiligo Extent Score for a Target Area (VESTA).
7. Physician Global Assessment for Extent (PGA extent).
8. Koebner’s Phenomenon in Vitiligo Score (K-VSCOR).
9. Vitiligo Signs of Activity Score (VSAS).
10. Potential Repigmentation Index (PRI).
11. Vitiligo Disease Improvement Score (VDIS).
12. Vitiligo Disease Activity Score (VDAS).

Detailed characteristics of these ClinROMs are provided in Supplementary Material S7.

All MPs and the corresponding QoEs for the included ClinROMs are summarized in Table 1 (first aim). Further clarifications and detailed results are available in Supplementary Material S9. The risk of bias assessment, which is a key factor in determining the QoE, is presented in Supplementary Material S8.

The results are organized based on the constructs measured:

- Extent/Repigmentation.
- Evolution/Activity.
- Composite Measures (Multiple Constructs).

Only (in)sufficient MP ratings supported by a moderate/high QoE are described in the result section below (second aim).

### 3.1. ClinROMs Measuring Extent/Repigmentation

The ClinROMs designed to assess extent/repigmentation include VASI, F-VASI, VES, VESplus, VESTA, PGA extent, and PRI.

- **VASI, VES, and VESplus**: These instruments evaluate disease extent as a percentage of affected body surface area (BSA) while accounting for the degree of depigmentation or repigmentation per body area. VES and VESplus rely on a picture-based scoring system. All three instruments achieved sufficient ratings for four MPs (inter- and intra-rater reliability, construct validity, and responsiveness), each supported by a moderate to high QoE.
- **F-VASI**: This tool specifically measures the extent of facial vitiligo using fingertip units. It received a sufficient rating for intra-rater reliability, supported by a high QoE.
- **VESTA**: VESTA assesses the percentage of repigmentation in a single target lesion between two time points. It received sufficient ratings for intra- and inter-rater reliability, both supported by a moderate QoE.
- **PGA extent**: PGA extent is a global measure of disease extent and is often used to complement other instruments. It was rated sufficient for convergent construct validity (moderate QoE) but insufficient for inter-rater reliability (moderate QoE).
- **PRI**: This instrument predicts the degree of repigmentation. However, no sufficient MP ratings supported by a moderate or high QoE were obtained.

### 3.2. ClinROMs Measuring Evolution/Activity

The ClinROMs assessing evolution (dynamic changes over time) or activity (based on assessment at a single time point—vitiligo activity signs) include VDIS, VDAS, VSAS, and K-VSCOR.

- **VDIS and VDAS**: These instruments measure improvement (repigmentation) or worsening (activity) across body regions. Both received sufficient ratings for four MPs, including inter- and intra-rater reliability as well as discriminant and convergent construct validity.
- **VSAS**: The VSAS quantifies clinical signs of activity and is the only validated ClinROM for this purpose. It received sufficient ratings for three MPs (inter-rater reliability, intra-rater reliability, and convergent construct validity), all supported by a moderate to high QoE.
- **K-VSCOR**: This tool maps Koebner’s phenomenon at trauma-exposed sites. However, it did not achieve any sufficient MP ratings supported by a moderate or high QoE.

### 3.3. ClinROM Measuring Multiple Constructs

The VETFa encompasses three subscales that address different aspects of vitiligo: extent, staging, and spreading.

- **VETFa-extent**: This subscale measures disease extent using the rule of nine and the assumption that the surface of one hand (including fingers) represents 1% BSA. It received sufficient ratings for three MPs (inter- and intra-rater reliability, responsiveness) with a moderate to high QoE.
- **VETFa-staging**: This subscale assesses the degree of depigmentation on a 0–4 scale across body regions. It received a sufficient rating for intra-rater reliability and an insufficient rating for inter-rater reliability, both supported by a moderate to high QoE.
- **VETFa-spreading**: This subscale evaluates disease progression, stability, or repigmentation across body regions. It received a sufficient rating for intra-rater reliability and an insufficient rating for inter-rater reliability, both supported by a moderate to high QoE.

## 4. Discussion

This review comprehensively examines the measurement properties (MPs) of ClinROMs in vitiligo, aiming to support the selection of suitable ClinROMs for both research and clinical practice. However, comparing or selecting ClinROMs based solely on these data is challenging for several reasons:

1. Incomplete Evaluation of MPs: Despite significant validation efforts, no ClinROM has undergone a complete evaluation of all MPs.
2. Indeterminate or Low QoE: Some ratings lacked clarity or had insufficient methodological quality.
3. Variability in Assessed MPs: There was notable variability in the types of MPs assessed, such as content validity for K-VSCOR versus reliability for VES.
4. Cross-Cultural Validity: None of the reviewed articles addressed cross-cultural validity, which evaluates whether translated or culturally adapted ClinROM questions are comparable to the original version.

In the category of vitiligo extent/repigmentation, VASI, VES, and VESplus demonstrated the highest number of sufficiently rated MPs (n = 4) with a moderate/high Quality of Evidence (QoE).

For evolution (comparing 2 time points; improvement and/or worsening), VDIS_15/60_ and VDAS_15/60_ each had the highest number of sufficiently rated MPs (n = 4).

For activity, assessed at 1 single time point, VSAS was the only validated ClinROM including different clinical signs of activity, and achieved three sufficiently rated MPs, based on two studies [16,25].

Content validity, important per COSMIN guidelines, was analyzed in only five studies and lacking a moderate/high Quality of Evidence due to insufficient methodological information. Furthermore, content validity analysis in the ClinROMs’ final stage, which holds more value compared to that of pre-final stages, was analyzed in just one study, which concerned VASI [8]. Comprehensibility, an aspect of content validity, was not analyzed in any of the studies. This could be attributed to the fact that ClinROMs are typically designed by physicians for physicians, with the underlying assumption that if the developers comprehend the ClinROM, it is reasonable to expect that the target audience will likewise find it understandable.

A high variation in Fitzpatrick skin types was observed in the included study populations, except for skin type 1, with this being most probably linked to less/not noticeable lesions in skin type 1 leading to fewer medical consultations. This also emphasizes the importance of vitiligo in darker skin types due to its higher visibility [29].

This review is an update of the systematic review conducted by Vrijman et al. in 2012 [30], which analyzed the MPs of ClinROMs, PROMs, and Observer reported Outcome Measuress (ObsROMs) in vitiligo. Concerning ClinROMs, Vrijman et al. analyzed VASI and VETFa. The VASI analysis carried out by Hamzavi et al. in 2004 [10], in which VASI scores were compared with scores from a non-validated instrument, namely ‘investigator and patient global assessment’, has been interpreted by Vrijman et al. as criterion validity. According to COSMIN guidelines, this is, however, categorized as construct validity. For ClinROMs, there are no gold standards, except for the more comprehensive ClinROMs compared to their shortened counterparts. Additionally, responsiveness was derived in the current review based on a more recent publication, which was not included in Vrijman’s review. Regarding VETFa, investigated by Taïeb et al. in 2007 [23], the QoE for measurement error was labeled as poor by Vrijman et al. However, it was not retained in this review because neither a minimal important difference (MID) nor a Smallest Detectable Change (SDC) was actually provided. Furthermore, it is noteworthy that Aydin et al.’s point-counting method from 2007 [31] was included in the review of Vrijman et al., categorized as a ClinROM. Nevertheless, van Geel et al.’s systematic review already thoroughly assessed the MPs of this tool, along with other image analysis systems [27]. To avoid redundancy while incorporating unique data, this specific instrument was excluded from the analysis in this review.

In 2019 an analysis of the quality of vitiligo severity measures was also undertaken by Peralta-Pedrero et al. [32] Although they yielded similar findings, it is noteworthy that their results did not consistently correspond to our review. This can be attributed to a less stringent adherence to the COSMIN guidelines by Peralta-Pedrero et al.

The MID can be used to assess measurement error; however, it is not considered an MP and therefore falls beyond the scope of this review. Additionally, feasibility and interpretability, which are crucial for ClinROM selection, are not addressed in this systematic review because they are not considered MPs either [33]. However, an evaluation of these aspects is included in a separate recently published paper [34].

### Limitations and Strengths

Firstly, no universally accepted guideline exists specifically for the analysis of ClinROMs. That is the reason why the COSMIN guidelines, which were initially designed for PROMs, have been used. These may have inherent limitations when utilized for ClinROMs. To address this, the analysis team established additional agreements, all approved by CT, complementing the COSMIN guidelines (see Supplementary Material S5). Furthermore, the lower limit of requiring only 25% of the study population to have the condition of interest (vitiligo) is rather low, increasing the potential for selection bias. An additional limitation of this review is its restriction to English-only publications. Furthermore, it is acknowledged that N.v.G. and R.S., contributors to article analysis, are also authors on several included papers, particularly those related to VES, VESplus, PGA extent, VSAS, and VDIS/VDAS [19,20,22,25,26,35]. We acknowledge that the total self-citation rate is high in this manuscript. However, since this is a systematic review, self-citations cannot be excluded solely to reduce the self-citation rate without compromising the integrity and rigor of the systematic approach.

Moreover, the systematic search was conducted four times consecutively (including three updates), with different reviewers each time. This approach may introduce subjective bias.

A key strength of this study is that the results were derived from analyses conducted in accordance with COSMIN guidelines and guided by the recommendations of methodology expert C.T., although input from multiple methodological experts would be preferred.

## 5. Conclusions

This review has evaluated the validation status of ClinROMs for vitiligo. A total of six ClinROMs were identified based on the highest number of sufficiently rated quality aspects across two construct categories. It is important to note that none of the evaluated ClinROMs has undergone a complete assessment of all the relevant MPs (reliability, responsiveness, and validity) including content validity, which is considered as an important MP. This systematic review serves as an intermediate step in the process of identifying core instruments for vitiligo, which should be based on the high-quality analysis of all the relevant MPs and feasibility aspects [33].

## Figures and Tables

**Table 1:** Summarized rating and Quality of Evidence per measurement property of ClinROMs.

Vitiligo-Specific ClinROMS												
Reference	Relevance	Comprehensiveness	Comprehensibility	Structural Validity	Internal Consistency	Inter-Rater Reliability	Intra-Rater Reliability	Measurement Error	Criterion Validity	Discriminative Construct Validity	Convergent Construct Validity	Responsiveness
**K-VSCOR (Koebner’s Phenomenon in Vitiligo Score)**												
Diallo et al., 2013 [7]		+ (dev)		?						?		
**Summarized rating**		**+**		**?**						**?**		
**Overall evidence**		**low**		**NA**						**NA**		
**VASI (Vitiligo Area and Severity Index)**												
Kitchen et al., 2022 [8]	? (final)											
Mogawer et al., 2020 [9]											+	
Hamzavi et al., 2004 [10]											?	+
Mehri et al., 2022 [11]						+	+					
Komen et al., 2015 [12]						+	+	?			+	+
Rosmarin et al., 2020 [13]							+			?	?	+
Pourang et al., 2023 [14]						+	+				?	
Kumar et al., 2024 [15]						+						
Youssef et al., 2023 [16]											+	+
**Summarized rating**	**?**					**+**	**+**	**?**		**?**	**+**	**+**
**Overall evidence**	**NA**					**high**	**moderate**	**NA**		**NA**	**high**	**high**
**F-VASI (Facial—Vitiligo Area and Severity Index)**												
Bae et al., 2022 [17]						+	+	?			?	
Mehri et al., 2022 [11]						+	+					
Rosmarin et al., 2020 [13]							+			?	?	+
Pourang et al., 2023 [14]						-	+					
Banerjee et al., 2023 [18]						-	+					
**Summarized rating**						**+/-**	**+**	**?**		**?**	**?**	**+**
**Overall evidence**						**moderate**	**high**	**NA**		**NA**	**NA**	**very low**
**VES (Vitiligo Extent Score)**												
Mogawer et al., 2020 [9]											+	
van Geel et al., 2018 [19]						+	+					+
van Geel et al., 2016 [20]						+	+	?			+	
Mehri et al., 2022 [11]						+	+					
Chaweekulrat et al., 2021 [21]						+		?			+	
**Summarized rating**						**+**	**+**	**?**			**+**	**+**
**Overall evidence**						**high**	**high**	**NA**			**high**	**moderate**
**VESplus (Vitiligo Extent Score Plus)**												
van Geel et al., 2018 [19]						+	+					+
van Geel et al., 2018 [22]						+	+	?			+	
Youssef et al., 2023 [16]											+	+
**Summarized rating**						**+**	**+**	**?**			**+**	**+**
**Overall evidence**						**high**	**high**	**NA**			**high**	**high**
**VETFa (Vitiligo European Task Force assessment)—subscales: VETFa-extent, VETFa-staging, VETFa-spreading**												
Komen et al., 2015 [12]						+ (VETFa-extent) - (VETFa-staging) - (VETFa-spreading)	+ (all subscales)	? (VETFa-extent)				+ (VETFa-extent)
Taïeb et al., 2007 [23]	? (all subscales)	? (all subscales)				- (VETFa-staging) - (VETFa-spreading)						
**Summarized rating**	**?** (all subscales)	**?** (all subscales)				**+** (VETFa-extent) **-** (VETFa-staging) **-** (VETFa-spreading)	**+** (all subscales)	**?** (VETFa-extent)				**+** (VETFa-extent)
**Overall evidence**	**NA** (all subscales)	**NA** (all subscales)				**high** (VETFa-staging and VETFA-spreading) **moderate** (VETFa-extent)	**moderate** (all subscales)	**NA** (VETFa-extent)				**moderate** (VETFa-extent)
**VESTA (Vitiligo Extent Score for a Target Area)**												
Bae et al., 2019 [24]						+	+	?			?	
**Summarized rating**						**+**	**+**	**?**			**?**	
**Overall evidence**						**moderate**	**moderate**	**NA**			**NA**	
**VSAS (Vitiligo Signs of Activity Score)**												
van Geel et al., 2020 [25]	? (dev)	? (dev)				+	+				+	
Youssef et al., 2023 [16]											?	?
**Summarized rating**	**?**	**?**				**+**	**+**				**+**	**?**
**Overall evidence**	**NA**	**NA**				**moderate**	**moderate**				**high**	**NA**
**PGA extent (Physician Global Assessment for Extent)**												
van Geel et al., 2019 [26]						-					+	
**Summarized rating**						**-**					**+**	
**Overall evidence**						**moderate**					**moderate**	
**VDIS 15 and 60 (Vitiligo Disease Improvement Score) and VDAS 15 and 60 (Vitiligo Disease Activity Score)**												
van Geel et al., 2022 [27]	? (dev)					+	+			+	+	
**Summarized rating**	**?**					**+**	**+**			**+**	**+**	
**Overall evidence**	**NA**					**moderate**	**moderate**			**moderate**	**high**	
**PRI (Potential Repigmentation Index)**												
Benzekri et al., 2013 [28]												?
**Summarized rating**												**?**
**Overall evidence**												**NA**

‘+’ = sufficient rating; ‘-’ = insufficient rating; ‘?’ = indeterminate rating; ‘dev’ stands for an analysis performed in the development phase of the ClinROM of interest. ‘final’ stands for an analysis performed in the definitive form of the ClinROM of interest. NA = Not applicable. None of the included articles examined cross-cultural validity, which is why this measurement property has been left out from the table ‘evidence and rating’ and S7 ‘risk of bias checklist’.

## Data Availability

Data extracted from the studies included in this systematic review are available in the text and the online Supplementary Material. More details on the quality analyses are included in the Supplementary Material. Specific details can be provided upon reasonable request to the corresponding author. The templates used for data collection are accessible through the COSMIN website.

## Supplementary Materials

The following supporting information can be downloaded at: https://www.mdpi.com/article/10.3390/jcm14082548/s1, S1: search strategy (flow diagram and search terms). S2: selection criteria. S3: Description of the COSMIN checklist measurement properties and standards for reporting each measurement property. S4: Analysis Method for Quality Measurement Properties following the COSMIN guideline. S5: Internal analysis Agreements. S6: Table study characteristics. S7: Table ClinROM characteristics. S8: Table: COSMIN Risk of Bias Checklist. S9: Clarification of Measurement Properties Quality assessment results. References [35,36,37,38,39,40,41] are cited in Supplementary Materials.
