# Supplementary material for: A Quality Analysis of the Measurement Properties of the Clinician-Reported Outcome Measures for Vitiligo and of the Studies Assessing Them: A Systematic Review
Source: J Clin Med. 2025 Apr 8;14(8):2548. doi: 10.3390/jcm14082548 (PMC12028335; doi:10.3390/jcm14082548)
Supplement: Supplementary file 1 [file jcm-14-02548-s001.zip › 37.0 ClinROM S1 kopie.pdf]

PRISMA 2020 flow diagram for new systematic reviews which included searches of databases and registers only

S1 search strategy

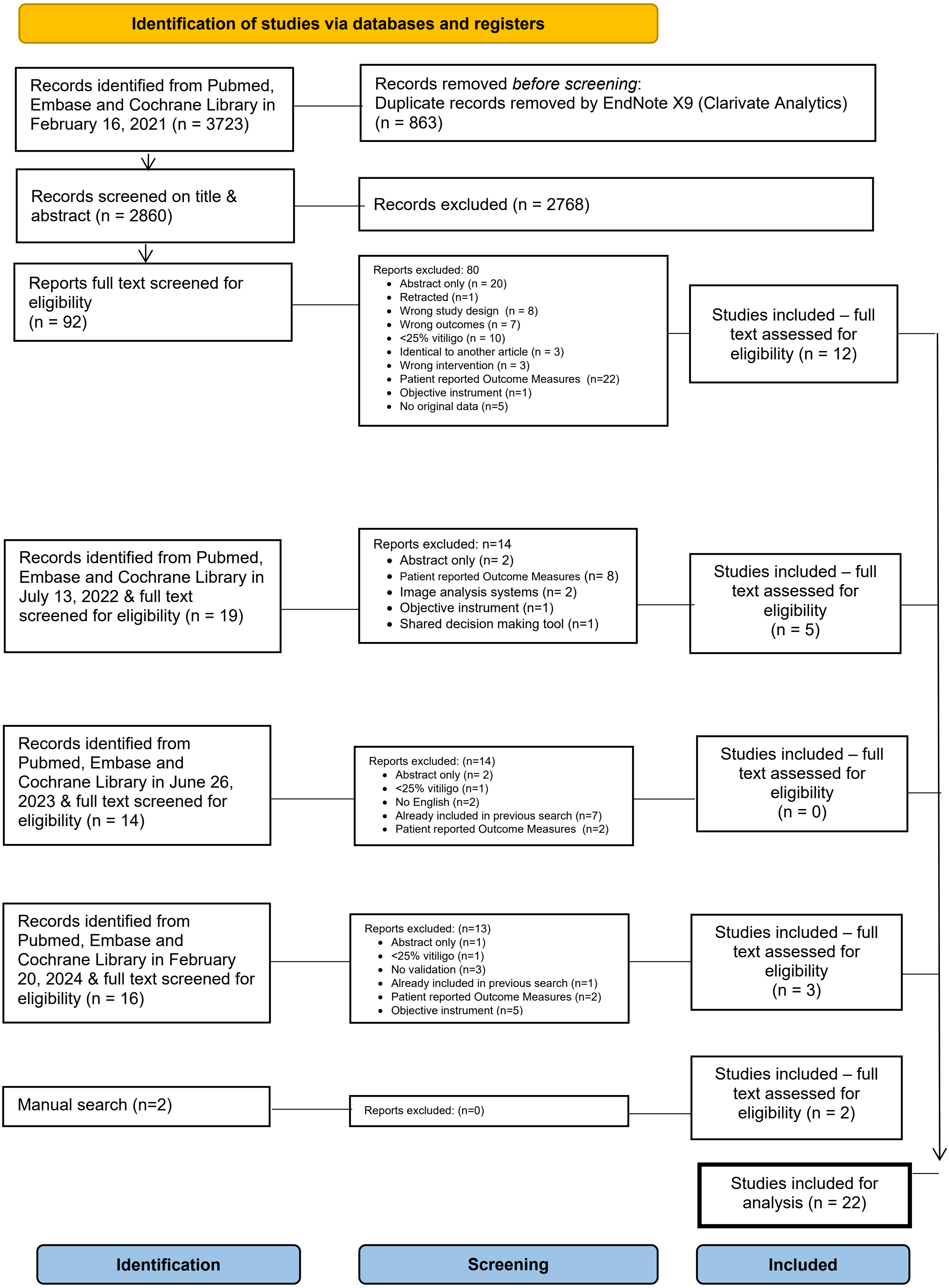

From: Page MJ, McKenzie JE, Bossuyt PM, Boutron I, Hoffmann TC, Mulrow CD, et al. The PRISMA 2020 statement: an updated guideline for reporting systematic reviews. BMJ 2021;372:n71. doi: 10.1136/bmj.n71

Search strategy

A literature search was supported by an experienced information specialist. References from relevant studies were screened manually for literature as well.

<u>Pubmed:</u>

Subject	lines	Search terms
Vitiligo	1	(“Vitiligo“[Mesh] OR “Vitiligo“[TIAB])
Clinimetric studies/ measurement properties filter (ref: Terwee CB)	2	(instrumentation[sh] OR methods[sh] OR "Validation Study"[pt] OR "Comparative Study"[pt] OR "psychometrics"[MeSH] OR psychometr*[TIAB] OR clinimetr*[tw] OR clinometr*[tw] OR "outcome assessment, health care"[MeSH] OR "outcome assessment"[TIAB] OR "outcome measure*"[tw] OR "observer variation"[MeSH] OR "observer variation"[TIAB] OR "Health Status Indicators"[Mesh] OR "reproducibility of results"[MeSH] OR reproducib*[TIAB] OR "discriminant analysis"[MeSH] OR reliab*[TIAB] OR unreliab*[TIAB] OR valid*[TIAB] OR "coefficient of variation"[TIAB] OR coefficient[TIAB] OR homogeneity[TIAB] OR homogeneous[TIAB] OR "internal consistency"[TIAB] OR (cronbach*[TIAB] AND (alpha[TIAB] OR alphas[TIAB])) OR (item[TIAB] AND (correlation*[TIAB] OR selection*[TIAB] OR reduction*[TIAB])) OR agreement[tw] OR precision[tw] OR imprecision[tw] OR "precise values"[tw] OR test-retest[TIAB] OR (test[TIAB] AND retest[TIAB]) OR (reliab*[TIAB] AND (test[TIAB] OR retest[TIAB])) OR stability[TIAB] OR interrater[TIAB] OR inter-rater[TIAB] OR intrarater[TIAB] OR intra-rater[TIAB] OR intertester[TIAB] OR inter-tester[TIAB] OR intratester[TIAB] OR intra-tester[TIAB] OR interobserver[TIAB] OR inter-observer[TIAB] OR intraobserver[TIAB] OR intra-observer[TIAB] OR intertechnician[TIAB] OR inter-technician[TIAB] OR intratechnician[TIAB] OR intra-technician[TIAB] OR interexaminer[TIAB] OR inter-examiner[TIAB] OR intraexaminer[TIAB] OR intra-examiner[TIAB] OR interassay[TIAB] OR inter-assay[TIAB] OR intraassay[TIAB] OR intra-assay[TIAB] OR interindividual[TIAB] OR inter-individual[TIAB] OR intraindividual[TIAB] OR intra-individual[TIAB] OR interparticipant[TIAB] OR inter-participant[TIAB] OR intraparticipant[TIAB] OR intra-participant[TIAB] OR kappa[TIAB] OR kappa's[TIAB] OR kappas[TIAB] OR repeatab*[tw] OR ((replicab*[tw] OR repeated[tw]) AND (measure[tw] OR measures[tw] OR findings[tw] OR result[tw] OR results[tw] OR test[tw] OR tests[tw])) OR generaliza*[TIAB] OR generalisa*[TIAB] OR concordance[TIAB] OR (intraclass[TIAB] AND correlation*[TIAB]) OR discriminative[TIAB] OR "known group"[TIAB] OR "factor analysis"[TIAB] OR "factor analyses"[TIAB] OR "factor structure"[TIAB] OR "factor structures"[TIAB] OR dimension*[TIAB] OR subscale*[TIAB]

		OR (multitrait[TIAB] AND scaling[TIAB] AND (analysis[TIAB] OR analyses[TIAB])) OR "item discriminant"[TIAB] OR "interscale correlation*"[TIAB] OR error[TIAB] OR errors[TIAB] OR "individual variability"[TIAB] OR "interval variability"[TIAB] OR "rate variability"[TIAB] OR (variability[TIAB] AND (analysis[TIAB] OR values[TIAB])) OR (uncertainty[TIAB] AND (measurement[TIAB] OR measuring[TIAB])) OR "standard error of measurement"[TIAB] OR sensitiv*[TIAB] OR responsive*[TIAB] OR (limit[TIAB] AND detection[TIAB]) OR "minimal detectable concentration"[TIAB] OR interpretab*[TIAB] OR ((minimal[TIAB] OR minimally[TIAB] OR clinical[TIAB] OR clinically[TIAB]) AND (important[TIAB] OR significant[TIAB] OR detectable[TIAB]) AND (change[TIAB] OR difference[TIAB])) OR (small*[TIAB] AND (real[TIAB] OR detectable[TIAB]) AND (change[TIAB] OR difference[TIAB])) OR "meaningful change"[TIAB] OR "ceiling effect"[TIAB] OR "floor effect"[TIAB] OR "item response model"[TIAB] OR irt[TIAB] OR rasch[TIAB] OR "differential item functioning"[TIAB] OR dif[TIAB] OR "computer adaptive testing"[TIAB] OR "item bank"[TIAB] OR "cross-cultural equivalence"[TIAB])
Exclusion filter: undesired publication types	3	("address"[Publication Type] OR "biography"[Publication Type] OR "case reports"[Publication Type] OR "comment"[Publication Type] OR "directory"[Publication Type] OR "editorial"[Publication Type] OR "festschrift"[Publication Type] OR "interview"[Publication Type] OR "lecture"[Publication Type] OR "legal case"[Publication Type] OR "legislation"[Publication Type] OR "letter"[Publication Type] OR "news"[Publication Type] OR "newspaper article"[Publication Type] OR "patient education handout"[Publication Type] OR "popular work"[Publication Type] OR "congress"[Publication Type] OR "consensus development conference"[Publication Type] OR "consensus development conference, nih"[Publication Type] OR "practice guideline"[Publication Type])
Exclusion filter: exclusion of non-human studies	4	("animals"[MeSH Terms] NOT "humans"[MeSH Terms])
Combination of concepts	5	(1 AND 2) NOT 3 NOT 4

Embase:

Subject	Lines	Search terms
Vitiligo	1	(‘Vitiligo’/exp OR ‘Vitiligo’:ti,ab,kw)

Clinimetric studies/ measurement properties filter (ref: Terwee CB)	2	('intermethod comparison'/exp OR 'data collection method'/exp OR 'validation study'/exp OR 'feasibility study'/exp OR 'pilot study'/exp OR 'psychometry'/exp OR 'reproducibility'/exp OR reproducib*:ab,ti OR 'audit':ab,ti OR psychometr*:ab,ti OR clinimetr*:ab,ti OR clinometr*:ab,ti OR 'observer variation'/exp OR 'observer variation':ab,ti OR 'discriminant analysis'/exp OR 'validity'/exp OR reliab*:ab,ti OR valid*:ab,ti OR 'coefficient':ab,ti OR 'internal consistency':ab,ti OR (cronbach*:ab,ti AND ('alpha':ab,ti OR 'alphas':ab,ti)) OR 'item correlation':ab,ti OR 'item correlations':ab,ti OR 'item selection':ab,ti OR 'item selections':ab,ti OR 'item reduction':ab,ti OR 'item reductions':ab,ti OR 'agreement':ab,ti OR 'precision':ab,ti OR 'imprecision':ab,ti OR 'precise values':ab,ti OR 'test-retest':ab,ti OR ('test':ab,ti AND 'retest':ab,ti) OR (reliab*:ab,ti AND ('test':ab,ti OR 'retest':ab,ti)) OR 'stability':ab,ti OR 'interrater':ab,ti OR 'inter-rater':ab,ti OR 'intrarater':ab,ti OR 'intra-rater':ab,ti OR 'intertester':ab,ti OR 'inter-tester':ab,ti OR 'intratester':ab,ti OR 'intratester':ab,ti OR 'interobeserver':ab,ti OR 'inter-observer':ab,ti OR 'intraobserver':ab,ti OR 'intraobserver':ab,ti OR 'intertechnician':ab,ti OR 'inter-technician':ab,ti OR 'intratechnician':ab,ti OR 'intratechnician':ab,ti OR 'interexaminer':ab,ti OR 'inter-examiner':ab,ti OR 'intraexaminer':ab,ti OR 'intraexaminer':ab,ti OR 'interassay':ab,ti OR 'inter-assay':ab,ti OR 'intraassay':ab,ti OR 'intra-assay':ab,ti OR 'interindividual':ab,ti OR 'inter-individual':ab,ti OR 'intraindividual':ab,ti OR 'intra-individual':ab,ti OR 'interparticipant':ab,ti OR 'inter-participant':ab,ti OR 'intraparticipant':ab,ti OR 'intraparticipant':ab,ti OR 'kappa':ab,ti OR 'kappas':ab,ti OR 'coefficient of variation':ab,ti OR repeatab*:ab,ti OR (replicab*:ab,ti OR 'repeated':ab,ti AND ('measure':ab,ti OR 'measures':ab,ti OR 'findings':ab,ti OR 'result':ab,ti OR 'results':ab,ti OR 'test':ab,ti OR 'tests':ab,ti)) OR generaliza*:ab,ti OR generalisa*:ab,ti OR 'concordance':ab,ti OR ('intraclass':ab,ti AND correlation*:ab,ti) OR 'discriminative':ab,ti OR 'known group':ab,ti OR 'factor analysis':ab,ti OR 'factor analyses':ab,ti OR 'factor structure':ab,ti OR 'factor structures':ab,ti OR 'dimensionality':ab,ti OR subscale*:ab,ti OR 'multitrait scaling analysis':ab,ti OR 'multitrait scaling analyses':ab,ti OR 'item discriminant':ab,ti OR 'interscale correlation':ab,ti OR 'interscale correlations':ab,ti OR ('error':ab,ti OR 'errors':ab,ti AND (measure*:ab,ti OR correlat*:ab,ti OR evaluat*:ab,ti OR 'accuracy':ab,ti OR 'accurate':ab,ti OR 'precision':ab,ti OR 'mean':ab,ti)) OR 'individual variability':ab,ti OR 'interval variability':ab,ti OR 'rate variability':ab,ti OR 'variability analysis':ab,ti OR ('uncertainty':ab,ti AND ('measurement':ab,ti OR 'measuring':ab,ti)) OR 'standard error of measurement':ab,ti OR

		sensitiv*:ab,ti OR responsive*:ab,ti OR (‘limit’:ab,ti AND ‘detection’:ab,ti) OR ‘minimal detectable concentration’:ab,ti OR interpretab*:ab,ti OR (small*:ab,ti AND (‘real’:ab,ti OR ‘detectable’:ab,ti) AND (‘change’:ab,ti OR ‘difference’:ab,ti)) OR ‘meaningful change’:ab,ti OR ‘minimal important change’:ab,ti OR ‘minimal important difference’:ab,ti OR ‘minimally important change’:ab,ti OR ‘minimally important difference’:ab,ti OR ‘minimal detectable change’:ab,ti OR ‘minimal detectable difference’:ab,ti OR ‘minimally detectable change’:ab,ti OR ‘minimally detectable difference’:ab,ti OR ‘minimal real change’:ab,ti OR ‘minimal real difference’:ab,ti OR ‘minimally real change’:ab,ti OR ‘minimally real difference’:ab,ti OR ‘ceiling effect’:ab,ti OR ‘floor effect’:ab,ti OR ‘item response model’:ab,ti OR ‘irt’:ab,ti OR ‘rasch’:ab,ti OR ‘differential item functioning’:ab,ti OR ‘dif’:ab,ti OR ‘computer adaptive testing’:ab,ti OR ‘item bank’:ab,ti OR ‘cross-cultural equivalence’:ab,ti)
Exclusion filter: undesired publication types	3	(‘case report’ OR ‘note’ OR ‘letter’ OR ‘editorial’):it
Exclusion filter: exclusion of non-human studies	4	(‘animal’/exp NOT ‘human’/exp)
Combination of concepts	5	(1 AND 2) NOT 3 NOT 4

Cochrane:

Subject	Linies	Search terms
Vitiligo	1	[mh “vitiligo”] OR vitiligo:ti,ab,kw
Clinimetric studies/ measurement properties filter (ref. terwee CB Development of a methodological	2	[mh “Instrumentation”] OR [mh “methods”] OR (“Validation Study”):pt OR (“Comparative Study”):pt OR [mh “psychometrics”] OR (psychometr*):ti,ab,kw OR (clinimetr*):ti,ab,kw OR (clinometr*):ti,ab,kw OR [mh “outcome assessment, health care”] OR (“outcome assessment”):ti,ab,kw OR (“outcome measure*”):ti,ab,kw OR [mh “observer variation”] OR (“observer variation”):ti,ab,kw OR [mh “Health Status Indicators”] OR [mh “reproducibility of results”] OR (reproducib*):ti,ab,kw OR [mh “discriminant analysis”] OR (reliab*):ti,ab,kw OR (“unreliab*”):ti,ab,kw OR (valid*):ti,ab,kw OR (“coefficient of variation”):ti,ab,kw OR coefficient:ti,ab,kw OR homogeneity:ti,ab,kw OR homogeneous:ti,ab,kw OR (“internal consistency”):ti,ab,kw OR ((cronbach*):ti,ab,kw AND (alpha:ti,ab,kw OR alphas:ti,ab,kw)) OR (item:ti,ab,kw AND

		((correlation*):ti,ab,kw OR (selection*):ti,ab,kw OR (reduction*):ti,ab,kw)) OR agreement:ti,ab,kw OR precision:ti,ab,kw OR imprecision:ti,ab,kw OR ("precise values"):ti,ab,kw OR test-retest:ti,ab,kw OR (test:ti,ab,kw AND retest:ti,ab,kw) OR ((reliab*):ti,ab,kw AND (test:ti,ab,kw OR retest:ti,ab,kw)) OR stability:ti,ab,kw OR interrater:ti,ab,kw OR inter-rater:ti,ab,kw OR intrarater:ti,ab,kw OR intra-rater:ti,ab,kw OR intertester:ti,ab,kw OR inter-tester:ti,ab,kw OR intratester:ti,ab,kw OR intra-tester:ti,ab,kw OR interobserver:ti,ab,kw OR inter-observer:ti,ab,kw OR intraobserver:ti,ab,kw OR intra-observer:ti,ab,kw OR intertechnician:ti,ab,kw OR inter-technician:ti,ab,kw OR intratechnician:ti,ab,kw OR intra-technician:ti,ab,kw OR interexaminer:ti,ab,kw OR inter-examiner:ti,ab,kw OR intraexaminer:ti,ab,kw OR intra-examiner:ti,ab,kw OR interassay:ti,ab,kw OR inter-assay:ti,ab,kw OR intraassay:ti,ab,kw OR intra-assay:ti,ab,kw OR interindividual:ti,ab,kw OR inter-individual:ti,ab,kw OR intraindividual:ti,ab,kw OR intra-individual:ti,ab,kw OR interparticipant:ti,ab,kw OR inter-participant:ti,ab,kw OR intraparticipant:ti,ab,kw OR intra-participant:ti,ab,kw OR kappa:ti,ab,kw OR (kappa's):ti,ab,kw OR kappas:ti,ab,kw OR (repeatab*):ti,ab,kw OR (((replicab*):ti,ab,kw OR repeated:ti,ab,kw) AND (measure:ti,ab,kw OR measures:ti,ab,kw OR findings:ti,ab,kw OR result:ti,ab,kw OR results:ti,ab,kw OR test:ti,ab,kw OR tests:ti,ab,kw)) OR (generaliza*):ti,ab,kw OR (generalisa*):ti,ab,kw OR concordance:ti,ab,kw OR (intraclass:ti,ab,kw AND (correlation*):ti,ab,kw) OR discriminative:ti,ab,kw OR ("known group"):ti,ab,kw OR ("factor analysis"):ti,ab,kw OR ("factor analyses"):ti,ab,kw OR ("factor structure"):ti,ab,kw OR ("factor structures"):ti,ab,kw OR (dimension*):ti,ab,kw OR (subscale*):ti,ab,kw OR (multitrait:ti,ab,kw AND scaling:ti,ab,kw AND (analysis:ti,ab,kw OR analyses:ti,ab,kw)) OR ("item discriminant"):ti,ab,kw OR ("interscale correlation*"):ti,ab,kw OR error:ti,ab,kw OR errors:ti,ab,kw OR ("individual variability"):ti,ab,kw OR ("interval variability"):ti,ab,kw OR ("rate variability"):ti,ab,kw OR (variability:ti,ab,kw AND (analysis:ti,ab,kw OR values:ti,ab,kw)) OR (uncertainty:ti,ab,kw AND (measurement:ti,ab,kw OR measuring:ti,ab,kw)) OR ("standard error of measurement"):ti,ab,kw OR (sensitiv*):ti,ab,kw OR (responsive*):ti,ab,kw OR (limit:ti,ab,kw AND detection:ti,ab,kw) OR ("minimal detectable concentration"):ti,ab,kw OR (interpretab*):ti,ab,kw OR ((minimal:ti,ab,kw OR minimally:ti,ab,kw OR clinical:ti,ab,kw OR clinically:ti,ab,kw) AND (important:ti,ab,kw

		OR significant:ti,ab,kw OR detectable:ti,ab,kw) AND (change:ti,ab,kw OR difference:ti,ab,kw)) OR ((small*):ti,ab,kw AND (real:ti,ab,kw OR detectable:ti,ab,kw) AND (change:ti,ab,kw OR difference:ti,ab,kw)) OR (“meaningful change”):ti,ab,kw OR (“ceiling effect”):ti,ab,kw OR (“floor effect”):ti,ab,kw OR (“item response model”):ti,ab,kw OR irt:ti,ab,kw OR rasch:ti,ab,kw OR (“differential item functioning”):ti,ab,kw OR dif:ti,ab,kw OR (“computer adaptive testing”):ti,ab,kw OR (“item bank”):ti,ab,kw OR (“cross-cultural equivalence”):ti,ab,kw
Exclusion filter: undesired publication types	3	(address:pt OR biography:pt OR (“case reports”):pt OR directory:pt OR editorial:pt OR festschrift:pt OR interview:pt OR lecture:pt OR (“legal case”):pt OR (“legislation”):pt OR (“news”):pt OR (“newspaper article”):pt OR (“patient education handout”):pt OR (“popular work”):pt OR (“comment”):pt OR (“directory”):pt OR (“editorial”):pt OR (“letter”):pt OR (“congress”):pt OR (“consensus development conference”):pt)
Exclusion filter: exclusion of non-human studies	4	([mh “animals”] NOT [mh “humans”])
bination of concepts	5	(1 AND 2) NOT 3 NOT 4
