# Supplementary material for: A Quality Analysis of the Measurement Properties of the Clinician-Reported Outcome Measures for Vitiligo and of the Studies Assessing Them: A Systematic Review
Source: J Clin Med. 2025 Apr 8;14(8):2548. doi: 10.3390/jcm14082548 (PMC12028335; doi:10.3390/jcm14082548)
Supplement: Supplementary file 1 [file jcm-14-02548-s001.zip › 37.0 ClinROM S2 kopie.pdf]

S2: Selection criteria

**Inclusion criteria**
ClinROMs<br>ClinROMs are defined in this review as instruments completed by a healthcare provider, which reports on a patient's health status.
If a minimum of 25% of the study population experiences segmental or non-segmental vitiligo, or if the evaluation of the measurement instrument's quality for the included vitiligo patients is conducted separately.
The quality assessment of at least one ClinROM measurement property is performed<br>OR<br>The development process of a ClinROM is discussed
Original research
Human subjects
Written in English

**Exclusion criteria**
Abstracts only
Control instruments*

ClinROM: Clinician-Reported Outcome Measure.
*Only clinimetric properties of the instruments of interest are included in this study
