# Supplementary material for: A Quality Analysis of the Measurement Properties of the Clinician-Reported Outcome Measures for Vitiligo and of the Studies Assessing Them: A Systematic Review
Source: J Clin Med. 2025 Apr 8;14(8):2548. doi: 10.3390/jcm14082548 (PMC12028335; doi:10.3390/jcm14082548)
Supplement: Supplementary file 1 [file jcm-14-02548-s001.zip › 37.0 ClinROM S3 kopie.pdf]

# S3: Description of the COSMIN checklist measurement properties and standards for reporting each measurement property

| Measurement property | Definition | Criteria for good measurement properties |
|---|---|---|
| Content validity | Content validity refers to the extent to which the CLINROM is relevant (the CLINROM should be relevant for the construct of interest within a specific population and context of use), comprehensible (the CLINROM should be understood by patients as intended), and comprehensive (no key aspects of the construct should be missing). These three aforementioned aspects are examined on two levels: during the development of the CLINROM and when the CLINROM has reached its final form. Content validity is strictly considered when these three aspects are investigated within the definitive version of the CLINROM, hence these results carry more weight compared to results from the development phase. | Relevance is rated sufficient if the items of the CLINROM are considered relevant to the construct of interest, the target population, and the context of use. Additionally, if the response options and recall period are appropriate.<br>Comprehensiveness is rated sufficient if all key concepts are included.<br>Comprehensibility is rated sufficient if the items, response options and instructions are understood by the target population as intended. |
| Structural validity | The degree to which the scores of a CLINROM are an adequate reflection of the dimensionality of the construct to be measured. | If the **Classical Test Theory** is used as a framework to measure structural validity, and CFA is used, the measurement should have a Comparative Fit Index (CFI), Tucker-Lewis Index (TLI) or a comparable measure which is greater than 0.95. Or the Root Mean Square Error of Approximation (RMSEA) should be less than 0.06, or the Standardized Root Mean Residual (SRMR) should be less than 0.082.<br><br>If the **Factor Item Response Theory (IRT) or Rasch analysis** is used the measurement should not violate unidimensionality. This means that the CFI, TLI, or a similar measure should be greater than 0.95, or the RMSEA should be less than 0.06, or the SRMR should be less than 0.08.<br>AND<br>The measurement should not violate local independence. This means that after considering the main factor, any remaining correlations between items (residual correlations) should be less than 0.20, or Q3's should be less than 0.37.<br>AND<br>The measurement should not violate monotonicity. Which means adequate looking graphs or item scalability, which should be greater than 0.30.<br>AND<br>The measurement model should show good fit. This means for IRT that the $\chi 2$ (chi-square) value should be greater than 0.01. If Rasch analysis is used, this means that the mean squares for infit and outfit should be between 0.5 and 1.5, or that the Z-standardized values should be greater than -2 and less than 2.<br><br>NB: Structural validity is only relevant for CLINROMs that are based on a reflective model, which is a model in which all items are a manifestation of the same underlying construct. Its counterpart is a formative model, in which the items together form the construct. These items do not need to be correlated. |
| Internal consistency | Internal consistency represents the degree of the interrelatedness among the items within a subscale. The subscales/dimensionality is defined by the structural validity. | At least low evidence for sufficient structural validity AND Cronbach's alpha(s) ≥ 0.70 for each unidimensional scale or subscale.<br><br>NB: Internal consistency is only relevant for CLINROMs that are based on a reflective model, which is a model in which all items are a manifestation of the same underlying construct. Its counterpart is a formative model, in which the items together form the construct. These items do not need to be correlated. |
| Reliability | The extent to which scores for patients who have not changed are the same for repeated measurement under several conditions: e.g. by different persons on the same occasion (inter-rater); or by the same persons (i.e. raters or responders) on different occasions (intra-rater) | Intrarater reliability should be calculated using Intraclass Correlation Coefficient (ICC) for continuous scores or kappa for dichotomous, ordinal or nominal scores. They should be equal to or greater than 0.70. |
| Measurement error | The systematic and random error of a patient's score that is not attributed to true changes in the construct to be measured | The Smallest Detectable Change (SDC) or Limits of Agreement (LoA) should be less than the Minimal Important Difference (MID). The MID should be calculated within a similar study population as the SDC. |
| Criterion validity | Criterion validity is defined as the degree to which the scores of a CLINROM are an adequate reflection of a "gold standard". No gold standards exist for CLINROMS, the only exception of a gold standard is when a shortened instrument | If the correlation with the gold standard is equal or greater than 0.70 or the Area Under the Curve is greater than or equal to 0.70 |

| | | |
|---|---|---|
| | is compared to the original long version. In that case, the original long version can be considered the gold standard. | |
| Construct validity | Construct validity is the degree to which the scores of a CLINROM are consistent with hypotheses. With regard to relationships to scores of other validated instruments (Convergent Validity) and differences between relevant groups (Discriminant validity) based on the assumption that the CLINROM validly measures the construct to be measured. | The result is in accordance with the hypothesis. The hypothesis can either be the one articulated by the authors of the paper or a generic hypothesis where instruments with similar constructs are expected to exhibit a correlation greater than 0.5, instruments with related but dissimilar constructs should have a correlation ranging from 0.3 to 0.5, and for unrelated constructs, the correlation should be less than 0.3. When no validation studies about the comparative instrument could be found in literature, the results of the analysis were not taken into account because the instrument is rendered unreliable. In terms of discriminative construct validity, meaningful changes (based on a logical rationale) should be observed between relevant subgroups. For instance, among patients with anticipated high versus low levels of the construct of interest. |
| Responsiveness | The ability of a CLINROM to detect change over time in the construct to be measured. | The outcome aligns with the proposed hypothesis, or the Area Under the Curve is greater than or equal to 0.70. It is important to know the magnitude and direction of change in order to make an accurate assessment of responsiveness. |
| Cross-cultural validity | Measures whether the performance of the questions on a translated or culturally adapted CLINROM are similar or comparable to the performance of the questions in the original version of the CLINROM. | No significant variations were discovered among group factors, such as age, gender, or language, in the multiple group factor analysis. Alternatively, there was no significant Differential Item Functioning observed for group factors, indicated by McFadden's $R^2$ being less than 0.02. |

Mokkink et al., 2018; Prinsen et al., 2018; Terwee et al., 2018[17, 22, 26]
