# Supplementary material for: A Quality Analysis of the Measurement Properties of the Clinician-Reported Outcome Measures for Vitiligo and of the Studies Assessing Them: A Systematic Review
Source: J Clin Med. 2025 Apr 8;14(8):2548. doi: 10.3390/jcm14082548 (PMC12028335; doi:10.3390/jcm14082548)
Supplement: Supplementary file 1 [file jcm-14-02548-s001.zip › 37.0 ClinROM S4 kopie.pdf]

S4: Analysis Method for Quality Measurement Properties following the COSMIN guideline

First, a risk-of-bias assessment for every study, adhering to the COSMIN risk-of-bias checklist[17, 22, 26] was assigned. This evaluation primarily scrutinizes the study's quality, encompassing elements such as the utilization of appropriate statistical analyses and accurate data handling (independently and codified). Consequently, a score is allocated to each study for each examined measurement property, spanning the spectrum from 'inadequate' to 'excellent.'

All results of the analysed measurement properties presented by the included studies are rated separately for each ClinROM using COSMIN's 'criteria of good measurement properties' [17, 22, 26]. For instance, internal consistency (one of the measurement properties) is deemed sufficient when Cronbach's alpha > 0.7. Further details can be found in Suppl. Mat. S3. Subsequently, the given ratings on the same MP within a particular ClinROM are consolidated. This aggregation follows the COSMIN guidelines and yields a summarized rating per measurement property per ClinROM.

From a rational perspective, a summarized rating based on a single study is less reliable than one derived from multiple studies of exceptional quality. Consequently, the trustworthiness of each summarized rating was evaluated by assessing the quality of evidence following the 'Grading of Recommendations, Assessment, Development, and Evaluation (GRADE) approach.' The quality of evidence considers various factors, including the risk-of-bias scores of the involved studies, the total number of enrolled patients and the consistency of individual ratings across studies.

Figure S1: 'Analysis Method for Quality Measurement Properties'

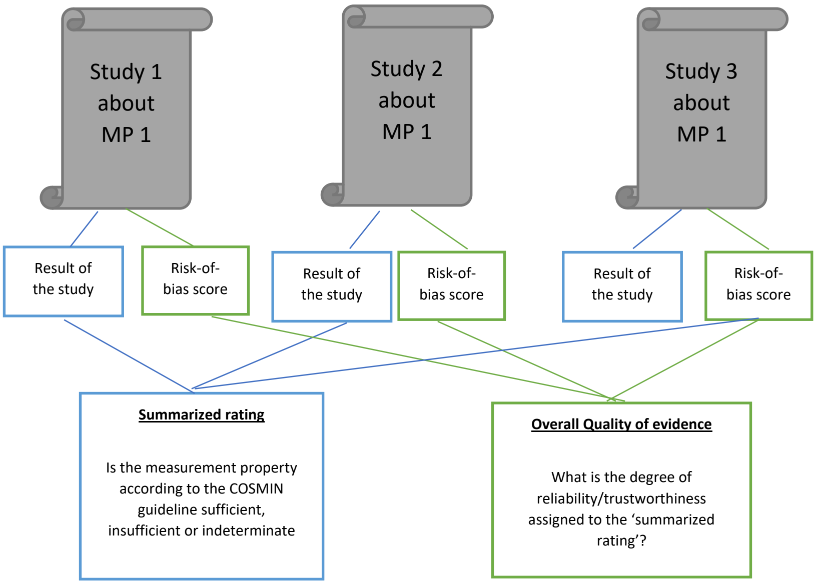

*MP = measurement property e.g., reliability
