# Supplementary material for: A Quality Analysis of the Measurement Properties of the Clinician-Reported Outcome Measures for Vitiligo and of the Studies Assessing Them: A Systematic Review
Source: J Clin Med. 2025 Apr 8;14(8):2548. doi: 10.3390/jcm14082548 (PMC12028335; doi:10.3390/jcm14082548)
Supplement: Supplementary file 1 [file jcm-14-02548-s001.zip › 37.0 ClinROM S5 kopie.pdf]

S5 Internal analysis Agreements

Below, all internal agreements can be found within the research team, supplementary to the COSMIN guidelines.

The primary challenge encountered during the ClinROM analysis was the fact that the COSMIN manual is primarily designed for evaluating the quality of PROMs rather than ClinROMs. Consequently, the criteria appeared to be excessively stringent for ClinROM assessment. To conduct a meaningful analysis, internal adaptations were made. For example, according to the COSMIN guideline, a "**total sample size**" of at least 100 is typically required to provide a robust quality of evidence. However, it was decided that the sample size would be considered sufficient if there were either at least 2 raters assessing 20 patients or 3 raters assessing 15 patients in total. This internal agreement was extended for a few articles at both extremes; more than 3 raters with fewer than 15 patients and fewer than 2 raters with more than 20 patients. For instance, in the study by Komen et al.[2] (VETFa) 33 patients were rated by a single rater. In the case of Bae et al.[1]'s (F-VASI), 11 patients were assessed by 6 physicians. Additionally, Mogawer et al.[3] evaluated VASI with a single rater assessing 101 patients.

Two additional criteria were included in the COSMIN guideline to assess **reliability** specifically for ClinROMs: "Did the professional(s) administer the measurement without prior knowledge of scores or values from previous measurements in the same patients?" and "Did the professional(s) assign scores or determine values without access to scores or values from other repeated measurements in the same patients?" Since these criteria were seldom explicitly mentioned in the included studies, an internal guideline was established. For intrarater reliability, with a minimum interval of

2 weeks between measurements, it was ensured that the raters were unaware of previous scores. Regarding interrater reliability, it was assumed that these criteria were met, as it was assumed that the raters were unaware of each other's scores unless there were specific reasons to believe otherwise. Regarding he following criterion for assessing intrarater reliability; "Were the measurement conditions similar for the measurements – except for the condition being evaluated as a source of variation?". It has been established internally that it is adequate to ascertain whether the patients were assessed live or from photographs on two separate occasions. This has been considered to represent similar conditions. Concerning **measurement error**, as per COSMIN guidelines, the Minimal Important Difference (MID) should align with the study population used for calculating the minimal detectable change. Significant variability within vitiligo populations makes it unsuitable to apply a MID calculated in a different population than that used for minimal detectable change[4]. Hence, MID should correspond to a similar study population concerning vitiligo extent as minimal detectable change. Unfortunately, many studies calculate minimal detectable change without concurrent assessment of MID in a similar population, hindering measurement error assessment.

In the evaluation of **content validity**, the assessment of risk of bias was conducted stringently, resulting in numerous 'doubtful' ratings due to the frequent absence of essential data in the respective articles. For example, in accordance with the COSMIN guidelines, articles should have explicitly documented whether patient interviews were guided by an appropriate interview protocol, if interviews were recorded and transcribed verbatim, and whether data was independently analyzed by at least two researchers. In the context of concept elicitation, one of the criteria stipulated that collected data should undergo independent coding, a requirement that was explicitly

fulfilled in only a few articles. To prevent rendering an inadequate score to nearly every article that conducted concept elicitation in vitiligo patients, the rating for this aspect was adjusted to 'not reported' rather than 'inadequate'.

Cross-cultural validity, which assesses the comparability of translated or culturally adapted ClinROM questions to their original version, was not addressed in any of the reviewed articles. Consequently, this measurement property has been excluded from both the 'evidence & rating' table and the 'risk-of-bias checklist' table.

In this current systematic review, the primary focus centers on the evaluation of measurement properties. Nevertheless, it is imperative to acknowledge that the COSMIN guidelines advocate for a comprehensive assessment that encompasses not only measurement properties but also the extraction of data related to **feasibility and interpretability**.

In accordance with the COSMIN guideline, it is stated that the **reviewers' team** (JD, LD, and SH) could also provide their own **opinion** on content validity (comprehensiveness, comprehensibility, relevance). Due to the limited clinical expertise of the team, it was decided not to take into account the personal opinions of the reviewer team in the analysis ratings.

References

1. Bae, J. M., et al. (2022). "Development and validation of the fingertip unit for assessing Facial Vitiligo Area Scoring Index." Journal of the American Academy of Dermatology **86**(2): 387-393.

2. Komen, L., et al. (2015). "Vitiligo Area Scoring Index and Vitiligo European Task Force assessment: reliable and responsive instruments to measure the degree of depigmentation in vitiligo." The British journal of dermatology **172**(2): 437-443.

3. Mogawer, R. M., et al. (2020). "Comparative analysis of the body surface area calculation method used in vitiligo extent score vs the hand unit method used in vitiligo area severity index." Journal of cosmetic dermatology **19**(10): 2679-2683.

4. Speeckaert, R., et al. (2022). "The Meaning and Reliability of Minimal Important Differences (MIDs) for Clinician-Reported Outcome Measures (ClinROMs) in Dermatology-A Scoping Review." Journal of personalized medicine **12**(7).
