# Supplementary material for: A Quality Analysis of the Measurement Properties of the Clinician-Reported Outcome Measures for Vitiligo and of the Studies Assessing Them: A Systematic Review
Source: J Clin Med. 2025 Apr 8;14(8):2548. doi: 10.3390/jcm14082548 (PMC12028335; doi:10.3390/jcm14082548)
Supplement: Supplementary file 1 [file jcm-14-02548-s001.zip › 37.0 ClinROM S6 kopie.pdf]

S6: Table study characteristics: vitiligo specific ClinROMs

**vitiligo specific ClinROMS**					
		**Age (*y*)**	**Gender**	**Affected BSA *(%)***	**Skin type or ethnicity *(n(%))***
**K-VSCOR (Koebner's phenomenon in vitiligo score)**					
Reference: Diallo et al., 2013 (10)					
Country	France	*mean, +-SD ,*<br>[*range*]<br>subgroup n=351:<br>25.75 +- 18.4, [2–74]<br>subgroup n=285:<br>23.00 +-16.32, [5–68]	subgroup n=351:<br>F: n=204 (58%)<br>M: n=147 (42%)<br>subgroup n=285:<br>F: n=164 (57.5%)<br>M: n=121 (42.5%)	NR	NR
Type of vitiligo	non-segmental				
Sample size *(n)*	n=351 (pt assessed using pre-final ClinROM by 2 ph)<br>n=285 (pt assessed using ClinROM by 3 ph)				
**VASI (Vitiligo Area and Severity Index)**					
Reference: Kitchen et al., 2022 (12)					
Country	USA (pt)<br>USA, India, Egypt (ph)	*mean (median)*<br>[*range*]<br>subgroup n=60:<br>34.5 (34) [12–62]	subgroup n=60:<br>F: n=38 (63%)<br>M: n=22 (37%)	*Mean (median) [range]*<br>subgroup n=60:<br>18.3 (7.5) [2.1–60.0]	subgroup n=60:<br>Caucasian or white 35 (58); Black or African American 8 (13); Hispanic, Latino, or Spanish origin 5 (8); Asian 5 (8); Other 7 (12)<br><br>I 9 (15); II 11 (18); III 14 (23); IV 12 (20); V 6 (10); VI 8 (13)
Type of vitiligo	non-segmental				
Sample size *(n)*	n=60 (pt interviews)<br>n=14 (ph interviews)				
Reference: Mogawer et al., 2020 (16)					
Country	Egypt		M: n=49 (48.5%)		

Type of vitiligo	Acrofacial 31 (29.0%); Focal 2 (1.9%); Generalized 51(47.7%); Multifocal asymmetrical 16(15.0%); Universalis 1(0.9%)	*Mean, Standard Deviation, Median, [range]*<br>35.69, ±14.82, 32.00, [18.00-80.00]	F: n=52 (51.5%)	*Mea,n Standard Deviation, Median, [range]*<br>9.37, ±13.88, 4.80, [0.10-81.45]	II 2(2%); III 44(43.6%); IV 51(50.5%); VI 4(4%)
Sample size *(n)*	n=101 (pt assessed using ClinROM)				
Reference: Hamzavi et al., 2004 (11)					
Country	Canada, US	*mean (SD) [range]*<br>47 (12.7) [23-77]	M: n=9<br>F: n=13	mean (SD) [range]<br>15.7 (8.9) [4-35.7]	II: 9; III 5; IV: 1; V: 7<br>White: 15; Indo-Pakistani: 6; Chinese 1
Type of vitiligo	NR				
Sample size *(n)*	n=22 (pt assessed using ClinROM)				
Reference: Mehri et al., 2022 (15)					
Country	Idem to data of van Mehri et al., 2022 for VES	Idem to data of van Mehri et al., 2022 for VES	Idem to data of van Mehri et al., 2022 for VES	Idem to data of van Mehri et al., 2022 for VES	Idem to data of van Mehri et al., 2022 for VES
Type of vitiligo	Idem to data of van Mehri et al., 2022 for VES				
Sample size *(n)*	Idem to data of van Mehri et al., 2022 for VES				
Reference: Komen et al., 2015 (13)					
Country	The Netherlands	*mean; median (range)*<br>subgroup n=31: 46,8; 46 (22–85)<br>subgroup n=27: 44.3; 44 (19–73)<br>subgroup n=33 46.5; 46 (18–85)	subgroup n=31:<br>M: n=15 (48%)<br>F: n=16 (52%)<br>subgroup n=27:<br>M: n=17 (63%)<br>F: n=10 (37%)<br>subgroup n=33<br>M: n=15 (46%)<br>F: n=18 (54%)	*mean; median (range) (measured with VASI)*<br>subgroup n=31: 8.4; 3.7 (06–372)<br>subgroup n=27: 6.1; 3.9 (03–276)<br>subgroup n=33: 9.6; 4.5 (06–372)	subgroup n=31: II 15 (48); III 5 (16); IV 11 (36)<br>subgroup n=27: II 5 (18); III 10 (37); IV 11 (41); V 1 (4)<br>subgroup n=33: II 16 (49); III 6 (18); IV 11 (33)
Type of vitiligo	Non-segmental				
Sample size *(n)*	n=31 (pt assessed once using ClinROM by 2 ph)<br>n=27 (pt assessed twice, with 2-week interval, using ClinROM by 1 ph)<br>n=33 (pt assessed twice, with 6-months interval, using ClinROM by 1 ph)				
Reference: Rosmarin et al., 2020 (23)					
Country	US	*mean (SD)*	subgroup n=157:	*mean (SD)*	subgroup n=157:

Type of vitiligo	segmental<br>non-segmental	subgroup n=157: 48,3 (12·9)	M: n=73 (46%)<br>F: n=84 (54%)	subgroup n=157: 22·05 (18·38)	White: n=132 (84%)<br>Black: n=14 (9%)<br>Asian: n=5 (3%)<br>Other: n=6 (4%)
Sample size *(n)*	n=157 (pt assessed once using ClinROM)<br>n=53 (pt considered stable in whom intrarater reliability was assessed)				
Reference: Pourang et al., 2023 (20)					
Country	US	*range*<br>*34 to 73*	M: 40%<br>F: 60%	NR	II 2 (20); III 4 (40); VI 3 (30); IV 1 (10)
Type of vitiligo	NR				
Sample size *(n)*	n=10 (pt image setes assessed twice using ClinROM by 12 ph, with 1 week interval)				
Reference: Kumar et al., 2023 (14) (letter to editor)					
Country	US	NR	NR	NR	NR
Type of vitiligo	NR				
Sample size *(n)*	n=10 (pt image setes assessed using ClinROM by 7 ph)				
Reference: Youssef et al., 2023 (38)					
Country	Egypt	*Mean ± SD (Range)* 29.6 ± 13.7 (18–61)	M: n=9 (30%)<br>F: n=21 (70%)	*Mean ± SD (Range) (measured* with VASI before start UVB treatment)<br>4.38 ± 4.11(0.33–15.85)	III 14 (46.7); IV 16 (53.3)
Type of vitiligo	non-segmental				
Sample size *(n)*	n=30 (pt image sets on UVB assessed twice using ClinROM by				
**F-VASI (Facial-Vitiligo Area and Severity Index)**					
Reference: Bae et al., 2022 (3)					
Country	South-Korea, USA (pt)	Children n=22<br>Adolescent n=12<br>Adult n=64	M: n=39<br>F: n=59	NR	Asian n=65; White n=14; South African n=12; Native American n=7
Type of vitiligo	NR				
Sample size *(n)*	n=98 (pt assessed using ClinROM)				

Reference: Mehri et al., 2022 (15)					
Country	Idem to data of van Mehri et al., 2022 for VES	Idem to data of van Mehri et al., 2022 for VES	Idem to data of van Mehri et al., 2022 for VES	Idem to data of van Mehri et al., 2022 for VES	Idem to data of van Mehri et al., 2022 for VES
Type of vitiligo	Idem to data of van Mehri et al., 2022 for VES				
Sample size *(n)*	Idem to data of van Mehri et al., 2022 for VES				
Reference: Rosmarin et al., 2020 (23)					
Country	Idem to data of van Rosmarin et al., 2020 for VASI	Idem to data of van Rosmarin et al., 2020 for VASI	Idem to data of van Rosmarin et al., 2020 for VASI	Idem to data of van Rosmarin et al., 2020 for VASI	Idem to data of van Rosmarin et al., 2020 for VASI
Type of vitiligo	Idem to data of van Rosmarin et al., 2020 for VASI				
Sample size *(n)*	Idem to data of van Rosmarin et al., 2020 for VASI				
Reference: Pourang et al., 2023 (20)					
Country	Idem to data of van Pourang et al., 2024 for VASI	Idem to data of van Pourang et al., 2024 for VASI	Idem to data of van Pourang et al., 2024 for VASI	Idem to data of van Pourang et al., 2024 for VASI	Idem to data of van Pourang et al., 2024 for VASI
Type of vitiligo	Idem to data of van Pourang et al., 2024 for VASI				
Sample size *(n)*	Idem to data of van Pourang et al., 2024 for VASI				
Reference: Banerjee et al., 2023 (4) (letter to editor)					
Country	NR	NR	NR	NR	NR
Type of vitiligo	non-segmental				
Sample size *(n)*	n=70 (pt image sets assessed twice using ClinROM by 2 ph, with 24 week interval)				
**VES (Vitiligo Extent Score)**					
Reference: Mogawer et al., 2020 (16)					
Country	Idem to data of Mogawer et al. 2020 for VASI	Idem to data of Mogawer et al. 2020 for VASI	Idem to data of Mogawer et al. 2020 for VASI	Idem to data of Mogawer et al. 2020 for VASI	Idem to data of Mogawer et al. 2020 for VASI
Type of vitiligo	Idem to data of Mogawer et al. 2020 for VASI				

Sample size *(n)*	Idem to data of Mogawer et al. 2020 for VASI				
Reference: van Geel et al., 2018 (28) (letter to editor)					
Country	Belgium *(presumably, not explicitly stated in article)*	NR	NR	NR	NR
Type of vitiligo	NR				
Sample size *(n)*	n=28 (pt image sets on cream-therapy assessed using ClinROM by 3 ph)<br>n=29 (pt image sets on UVB therapy assessed using ClinROM by 2 ph)				
Reference: van Geel et al., 2016 (32)					
Country	the Netherlands<br>Belgium	*Mean*<br>subgroup n=31: 32.1<br>subgroup n=20: 42.3<br>subgroup n=10: 42.8	subgroup n=31:<br>M: 11<br>F: 20<br>subgroup n=20<br>M: 9<br>F: 11<br>subgroup n=10:<br>M: 5<br>F: 5	*Mean (range*)<br>subgroup n=31: 3.65 (0.02-58.72)<br>subgroup n=20: 2.17 (0.01-34.34)<br>subgroup n=10: 4.48 (4.48-63.76)	subgroup n=31: II: 4; III: 21; IV: 5; V: 1<br>subgroup n=20: II: 1; III: 6; IV: 11; V: 1; VI: 1<br>subgroup n=10: II: 1; III: 7; V: 2
Type of vitiligo	non-segmental				
Sample size *(n)*	n=31 (pt-image sets assessed using ClinROM by 11 raters)<br>n=20 (pt live and image sets assessed using ClinROM by 6 ph, image sets were assessed twice, with 2-week interval)<br>n=10 (pt-image sets assessed twice with 2-week interval using ClinROM by 5 ph)				
Reference: Mehri et al., 2022 (15) (letter to editor)					
Country	NR	NR	NR	NR	NR
Type of vitiligo	NR				

Sample size *(n)*	n=17 (pt image sets assessed using ClinROM by 7 ph)<br>n=10 (pt image sets of subgroup n=17 re-assessed with 6-month interval by 7ph)				
Reference: Chaweekulrat et al., 2021 (6)					
Country	Thailand	*median (range)*<br>42 (18–84)	M: 23 (23.0%)<br>F: 77 (77.0%)	*median (IQR)*<br>0.89 (0.44–3.02)	III 25 (25.0); IV 59 (59.0); V 16 (16.0)
Type of vitiligo	segmental, non-segmental				
Sample size *(n)*	n=100 (pt assessed using ClinROM by 2 ph)				
**VESplus (Vitiligo Extent Score Plus)**					
Reference: van Geel et al., 2018 (28)					
Country	Idem to data of van Geel et al. 2018 for VES	Idem to data of van Geel et al. 2018 for VES	Idem to data of van Geel et al. 2018 for VES	Idem to data of van Geel et al. 2018 for VES	Idem to data of van Geel et al. 2018 for VES
Type of vitiligo	Idem to data of van Geel et al. 2018 for VES				
Sample size *(n)*	Idem to data of van Geel et al. 2018 for VES				
Reference: van Geel et al., 2018 (36) (letter to editor)					
Country	the Netherlands<br>Belgium	NR	NR	NR	NR
Type of vitiligo	NR				
Sample size *(n)*	n=28 (pt on cream therapy image sets assessed using ClinROM)<br>n=29 (pt receiving UVB therapy image sets assessed using ClinROM)				
Reference: Youssef et al., 2023 (38)					
Country	Idem to data of Youssef et al., 2023 for VASI	Idem to data of Youssef et al., 2023 for VASI	Idem to data of Youssef et al., 2023 for VASI	Idem to data of Youssef et al., 2023 for VASI	Idem to data of Youssef et al., 2023 for VASI
Type of vitiligo	Idem to data of Youssef et al., 2023 for VASI				

Sample size *(n)*	Idem to data of Youssef et al., 2023 for VASI				
**VETFa (Vitiligo European Task Force assessment)**					
Reference: Komen et al., 2015 (13)					
Country	Idem to data of Komen et al. 2014 for VASI	Idem to data of Komen et al. 2014 for VASI	Idem to data of Komen et al. 2014 for VASI	Idem to data of Komen et al. 2014 for VASI	Idem to data of Komen et al. 2014 for VASI
Type of vitiligo	Idem to data of Komen et al. 2014 for VASI				
Sample size *(n)*	Idem to data of Komen et al. 2014 for VASI				
Reference: Taïeb et al., 2007 (25)					
Country	Italy, Spain, France, Germany, Belgian, Sweden, England (pt)	median: 40<br>missing n=5 (2.3%)<br><25: n=34 (15.7%)<br>[25–50]: n=131 (60.3%)<br>>50: n=47 (21.7%)	F: 146 (67.3)<br>M: 71 (32.7)	Not explicitly reported (graph given which shows predominantly BSA <5%, exact percentages not extractable)	Missing 7 (3.2)<br>I–II: 37 (17.0); III: 130 (60.0); IV: 43 (19.8)
Type of vitiligo	NR				
Sample size *(n)*	n=217 (pt assessed using ClinROM by 10 ph)				
**VESTA (Vitiligo Extent Score for a Target Area)**					
Reference: Bae et al., 2019 (2)					
Country	NR	NR	NR	NR	NR
Type of vitiligo	NR				
Sample size *(n)*	n=17 (pt image-sets assessed twice, with 2-week interval using CLinROM by 65 ph)				
**VSAS (Reliability and validity of the Vitiligo Signs of Activity Score)**					
Reference: van Geel et al., 2020 (33)					
Country	Europe (ph)<br>Belgium (pt)	*mean, median (range)*<br>subgroup n=23<br>36, 40 (17–64)	subgroup n=23<br>F: 12<br>M: 11	*mean, median (range)*<br>subgroup n=23<br>5.95, 2.28 (0·13–42·7)	subgroup n=23<br>II: 2; III: 19, IV: 1, V: 1
Type of vitiligo	non-segmental				
Sample size *(n)*	n=28 (ph involved in concept elicitation)				

	n=23 (pt image-sets assess twice with 2-month interval by 4 ph (first assessment) and 3 ph (second assessment) using ClinROM)				
Reference: Youssef et al., 2023 (38)					
Country	Idem to data of Youssef et al., 2023 for VASI	Idem to data of Youssef et al., 2023 for VASI	Idem to data of Youssef et al., 2023 for VASI	Idem to data of Youssef et al., 2023 for VASI	Idem to data of Youssef et al., 2023 for VASI
Type of vitiligo	Idem to data of Youssef et al., 2023 for VASI				
Sample size *(n)*	Idem to data of Youssef et al., 2023 for VASI				
**PGA extent (Physician Global assessment for Extent)**					
Reference: van Geel et al., 2019 (35)					
Country	Europ, Asia, Afria, North America, South America (ph)<br>Belgium (pt)	NR	NR	NR	II:2; III: 13, IV: 1; V: 1
Type of vitiligo	non-segmental				
Sample size *(n)*	n=20 (pt image-set assessed by 28 ph using ClinROM)				
**VDIS 15 & 60 (Vitiligo Disease Improvement Score) and VDAS 15 & 60 (Vitiligo Disease Activity Score)**					
Reference: van Geel et al., 2022 (29)					
Country	Belgium	*mean, median*<br>37, 39	F: 57.6%<br>M: 42.4%	NR	II: 14 (21.2); III: 39 (59.1); IV: 2 (3.0); V: 1 (1.5); VI: 1 (1.5); unknown: 9 (13.6)
Type of vitiligo	non-segmental				
Sample size *(n)*	n=66 (pt image sets assessed twice with [4-14] month-interval using ClinROM by 7 ph (first assessment) and 5 ph (second assessment)				
**PRI (potential repigmentation index)**					
Reference: Benzekri et al., 2013 (5) (letter to editor)					
Country	France	*mean*	M: n=18	>10% BSA: n=18	NR

Type of vitiligo	non-segmental	36.8	F: n=12	<10% BSA: n=12	
Sample size *(n)*	n=30 (pt assessed twice, with 6-month interval, using ClinROM)				
