# Supplementary material for: A Quality Analysis of the Measurement Properties of the Clinician-Reported Outcome Measures for Vitiligo and of the Studies Assessing Them: A Systematic Review
Source: J Clin Med. 2025 Apr 8;14(8):2548. doi: 10.3390/jcm14082548 (PMC12028335; doi:10.3390/jcm14082548)
Supplement: Supplementary file 1 [file jcm-14-02548-s001.zip › 37.0 ClinROM S7 kopie.pdf]

S7: Table ClinROM characteristics

Reference	Construct	Intended context of use	Number of subscales/dimensions *(n)*	Number of items *(n)*	Recall period	Response options	Total score range	Language
**K-VSCOR (Koebner's phenomenon in vitiligo score)**								
Diallo et al., 2013 (10)	The likelihood to have Koebner Phenomenon in vitiligo	clinical practice	Presumable 1, not explicitly stated in article	8 (disease duration), 6 (body areas)	present	0-1	0-56	English
**VASI (Vitiligo Area and Severity Index)**								
Kitchen et al., 2022 (12)<br>Mogawer et al., 2020 (16)<br>Hamzavi et al., 2004 (11)<br>Mehri et al., 2022 (15)<br>Komen et al., 2015 (13)	affected body surface area in vitiligo	clinical trials<br>clinical practice	Presumable 1, not explicitly stated in article	NA	present	0-100%	0-100%	English
**F-VASI (Facial-Vitiligo Area and Severity Index)**								
Bae et al., 2022 (3)<br>Mehri et al., 2022 (15)	extent of facial involvement in vitiligo patients	clinical trials<br>clinical practice	Presumable 1, not explicitly stated in article	NA	present	0-100%	0-100%	English
**VES (Vitiligo Extent Score)**								
Mogawer et al., 2020 (16)<br>van Geel et al., 2018 (28)<br>van Geel et al., 2016 (32)	vitiligo extent	clinical practice<br>clinical trials	1	19 body areas, selecting most resembling image	present	0-100%	range: 0-100% per area<br>total score: 0- 98.5	English

Mehri et al., 2022 (15)<br>Chaweekulrat et al., 2021 (6)								
**VESplus (Vitiligo Extent Score Plus)**								
van Geel et al., 2018 (28)<br>van Geel et al., 2018 (36)	assessment of extent and perifollicular repigmentation	NR	2<br>extent<br>repigmentation	19 body areas, selecting most resembling image and perifollicular repigmentation	present	0-100%	repigmentation: 5-90<br>range: 0-100% per area<br>total score: 0- 98.5	English
**VETFa (Vitiligo European Task Force assessment)**								
Komen et al., 2015 (13)<br>Taïeb et al., 2007 (25)	degree of depigmentation in vitiligo	clinical trials (1)	3:<br>area<br>staging<br>spreading	5 body areas rated for each subscale	present	area: %<br>staging of largest macula per body area and total: [0-4]<br>spreading per body area and total: [-1 – 1]	area: 0-100<br>staging 0-20<br>spreading -5 – 5	English
**VESTA (Vitiligo Extent Score for a Target Area)**								
Bae et al., 2019 (2)	repigmentation rate in target lesion	clinical practice<br>retrospective studies	3<br>locations of vitiligo<br>locations with improvement<br>locations with worsening	1. Estimating fully repigmented area<br>2. Estimating depigmented area<br>3. Estimating Perifollicular repigmentation	present	0-100%	0-100	English
**VSAS (Reliability and validity of the Vitiligo Signs of Activity Score)**								
van Geel et al., 2020 (33)	assessment of clinical activity signs	clinical trials<br>daily practice	3	15 body areas, grading activity signs	present	yes-no	0-15	English

			Number of locations with confetti-like depigmentation<br>Number of locations with Koebner phenomenon type 2B<br>Number of locations with hypochromic areas/borders			grade 1-2-3 (reflecting the intensity per area)		
**PGA extent (Physician Global Assessment for Extent)**								
van Geel et al., 2019 (35)	disease extent	standardizing definitions of inclusion criteria for clinical trials, epidemiological profiling of populations, and define global disease evolution over time	1	1	present	5-point scale	presumable 1-5, but not explicitly stated in article)	presumably English, but not specifically stated in article
**VDIS 15 & 60 (Vitiligo Disease Improvement Score) and VDAS 15 & 60 (Vitiligo Disease Activity Score)**								
van Geel et al., 2022 (34)	clinical changes in depigmentation over time (activity and improvement)	clinical practice<br>clinical trials	3<br>areas involved<br>improvement<br>worsening	15 body areas, grading clinical changes	present	-4 - 4	VDIS15, VDAS 15: 0-15<br>VDAS60, VDIS60: 0-60	English<br>Dutch
**PRI (potential repigmentation index)**								
Benzekri et al., 2013 (5)	potential repigmentation	clinical practice	1	4<br>1. number of lesions of at least 10 cm2 with pigmented hair and minimal pigmentation	present	(number of type 1 lesions + number of type 2 lesions) divided by (number of type 3 lesions +	0 - ∞	Presumably French, not reported explicitly in article

				2. number of lesions of at least 10 cm2 without pigment, but with pigmented hair<br>3. number of lesions of at least 10 cm2 without pigment and depigmented hair<br>4. number of lesions of at least 10 cm2 without hair and without pigment		number of type 4 lesions)		
