# Supplementary material for: A Quality Analysis of the Measurement Properties of the Clinician-Reported Outcome Measures for Vitiligo and of the Studies Assessing Them: A Systematic Review
Source: J Clin Med. 2025 Apr 8;14(8):2548. doi: 10.3390/jcm14082548 (PMC12028335; doi:10.3390/jcm14082548)
Supplement: Supplementary file 1 [file jcm-14-02548-s001.zip › 37.0 ClinROM S8 kopie.pdf]

S8:Table : COSMIN Risk of Bias Checklist: methodological quality of the methods used to assess the measurement properties of vitiligo specific ClinROMs

Reference	Relevance	Comprehensiveness	Comprehensibility	Structural validity	Internal consistency	Intrarater reliability	Interrater reliability	Measurement error	Criterion Validity	Discriminant construct validity	Convergent construct validity	Responsiveness
**K-VSCOR (Koebner's phenomenon in vitiligo score)**												
Diallo et al., 2013 (10)		Doubtful (dev)		Adequate						Doubtful		
**VASI (Vitiligo Area and Severity Index)**												
Kitchen et al., 2022 (12)	Doubtful (final)											
Mogawer et al., 2020 (16)											Adequate	
Hamzavi et al., 2004 (11)											Inadequate	Adequate
Mehri et al., 2022 (15)						Adequate	Adequate					
Komen et al., 2015 (13)						Doubtful	Adequate	Doubtful *(based on intrarater reliability data)* Adequate *(based on*			Adequate	Very good

								interrater reliability data)				
Rosmarin et al., 2020 (23)						Doubtful				Inadequate	Inadequate	Inadequate
Pourang et al., 2023 (20)						Doubtful	Doubtful				Inadequate	
Kumar et al., 2023 (14)							Adequate					
Youssef et al., 2023 (38)											Very good	Very good
**F-VASI (Facial-Vitiligo Area and Severity Index)**												
Bae et al., 2022 (3)						Adequate	Adequate	Adequate *(based on intrarater reliability data)*			Inadequate	
Mehri et al., 2022 (15)						Adequate	Adequate					
Rosmarin et al., 2020 (23)						Doubtful				Inadequate	Inadequate	Inadequate
Pourang et al, 2023 (20)						Doubtful	Doubtful					
Banerjee et al., 2023 (4)						Adequate	Adequate					
**VES (Vitiligo Extent Score)**												

Mogawer et al., 2020 (16)											Adequate	
van Geel, Bekkenk et al., 2018 (28)						Adequate	Adequate					Adequate
van Geel et al., 2016 (32)						Adequate	Adequate	Adequate *(based on interrater reliability data)* Adequate *(based on intrarater reliability data)*			Very good	
Mehri et al., 2022 (15)						Doubtful	Doubtful					
Chaweekulrat et al., 2021 (6)							Adequate	Adequate *(based on interrater reliability data)*			Very good	
**VESplus (Vitiligo Extent Score Plus)**												
van Geel, Bekkenk et al., 2018 (28)						Adequate	Adequate					Adequate
van Geel, Wolkerstorfer et al., 2018 (36)						Adequate	Adequate	Adequate *(based on interrater reliability data)* Adequate *(based on*			Very good	

								intrarater reliability data)				
Youssef et al., 2023 (38)											Very good	Very good
**VETFa (Vitiligo European Task Force assessment)**												
Komen et al., 2015 (13)						Doubtful	Adequate	Doubtful *(based on intrarater reliability data)* Adequate *(based on interrater reliability data)*				Adequate
Taïeb et al., 2007 (25)	Doubtful (dev)	Doubtful (dev)					Adequate					
**VESTA (Vitiligo Extent Score for a Target Area)**												
Bae et al., 2019 (2)						Adequate	Adequate	Adequate *(based on interrater reliability data)* Adequate *(based on intrarater reliability data*			Inadequate	
**VSAS (Reliability and validity of the Vitiligo Signs of Activity Score)**												
van Geel et al., 2020 (33)	doubtful (dev)	doubtful (dev)				Adequate	Adequate				Very good	
Youssef et al., 2023 (38)											Inadequate	Inadequate
**PGA extent (Physician Global Assessment for Extent)**												

van Geel et al., 2019 (35)							Adequate				Adequate	
**VDIS 15 & 60 (Vitiligo Disease Improvement Score) and VDAS 15 & 60 (Vitiligo Disease Activity Score)**												
van Geel et al., 2022 (34)	Doubtful (dev)	Doubtful (dev)	Doubtful (dev)			Adequate	Adequate			Adequate	Very good	
**PRI (Potential Repigmentation Index)**												
Benzekri et al., 2013 (5)												Very low

‘dev’ stands for an analysis performed in the development phase of the ClinROM of interest. ‘final’ stands for an analysis performed in the definitive form of the ClinROM of interest.

None of the included articles examined
