# Supplementary material for: A Quality Analysis of the Measurement Properties of the Clinician-Reported Outcome Measures for Vitiligo and of the Studies Assessing Them: A Systematic Review
Source: J Clin Med. 2025 Apr 8;14(8):2548. doi: 10.3390/jcm14082548 (PMC12028335; doi:10.3390/jcm14082548)
Supplement: Supplementary file 1 [file jcm-14-02548-s001.zip › 37.0 ClinROM S9 kopie.pdf]

S9: Clarification of Measurement Properties Quality assessment results

1. Quality assessment of Measurement Properties of VASI (Vitiligo Area Scoring Index)

Regarding **content validity**, relevance was assessed[12]. Eight clinicians perceived VASI as a valid framework for assessing vitiligo. Additionally, 60 patients were interviewed about each item; the affected surface area and the level of pigmentation, to determine if these "measured their priorities," as stated by the authors. However, relevance was rated as indeterminate because the appropriateness of response options and the recall period was not separately examined. This assessment comes with a moderate quality of evidence. The downgrading can primarily be attributed to insufficient information provided about the methodology.

Convergent construct validity received a sufficient overall rating with a high quality of evidence. This result is based on the following results and articles. Convergent construct validity was examined by comparing VASI to a ClinROM with a similar construct (VASI vs. VES: r=0.909[16]). Additionally two sub-analyses were performed where they took into account correlations among patients with vitiligo in areas not scored by VES (r=0.945[16]) and correlations among patients with vitiligo only in areas presented by VES (r=0.921[16]). The correlation between VASI and VESplus was also investigated, both before (r = 0.936) and after (r = 0.970) a UVB treatment course[38]. Additionally, Komen et al.[13] investigated the correlation between SA-VASI (the patient-reported outcome version of VASI) and VASI, which was very high (VASI vs. SA-VASI r=0.97). Hamzavi et al.[11] and Rosmarin et al.[23] assessed the correlation between VASI and an instrument considered as unvalidated, referred to as the 'investigator and

patient global assessment' in Hamzavi et al. and ‘Physician Global Vitiligo Assessment’ in Rosmarin et al., leading to 'indeterminate' ratings for this MP. An ICC between VASI and ImageJ, a validated method for area calculation, was computed for vitiligo on the hands[20]. However, ICC is not a measure of correlation but rather a measure of variability. This renders the results non-interpretable, resulting in an indeterminate rating.

Discriminative construct validity was mentioned in the study by Rosmarin et al.[23], wherein the study population was categorized into mild, moderate, and severe disease groups based on their 'Physician Global Vitiligo Assessment' scores. Subsequently, p-values were calculated, revealing a significant difference in VASI scores among the groups. However, no correlation was reported, which is essential for making an assertion regarding construct validity. Subsequently, an ‘indeterminate’ rating was given for this MP.

Responsiveness received a sufficient rating with a high quality of evidence based on the results of 4 studies. Hamzavi et al.[11] examined treated body parts versus the contra-lateral untreated side, as well as differences among various treated body parts. They observed a greater decrease in the VASI score for the treated body parts, aligning with the logical rationale that UVB treatment leads to repigmentation. It was also anticipated that there would be less repigmentation on the hands and feet compared to other body parts, which was supported by the analysis results. Komen et al. also investigated responsiveness, measuring 23% repigmentation with VASI after 6.3 months, in line with the authors' hypothesis of expecting >15% repigmentation (Komen et al., 2015). They explored the correlation between the percentage of repigmentation measured by the physician and a global assessment of improvement

(both patient and physician). However, these are non-validated instruments. On the other hand, the authors assumed that the face would show >10% more repigmentation than the hands, which was indeed the case, with 42% repigmentation for the hands and 0% for the face, calculated using VASI. Rosmarin et al.[23] also measured responsiveness for VASI. This after 24 weeks, where the correlation with the 'Patient Global Impression of Change–Vitiligo' (PaGIC-V) scores was calculated (correlation coefficient=0.44). PaGIC-V is considered to be an unvalidated instrument. The authors did not propose a hypothesis. However, this correlation aligns with the generic hypothesis suggested in the COSMIN guideline, which presumes that the correlation between related but dissimilar constructs should fall between 0.3 and 0.5. Youssef et al.[38] investigated the correlation between the change in VASI score before and after a UVB treatment course compared to VESplus score (r = 0.723). This aligns with a logical rationale that VESplus will decrease in proportion to the VASI score.

Inter- and intrarater reliability both received a sufficient rating (both ICC=0.93, as published by Komen et al.[13]; intrarater reliability: ICC=0.86 and interrater reliability: ICC=0.85 reported by Mehri et al., 2022[15]; intrarater reliability: ICC=0.99 reported by Rosmarin et al.[23]; interrater reliability (calculated twice with an interval of 7 days): ICC= 0.843 and ICC= 0.837 and intrarater reliability calculated 12 times: ICC=[0.869-0.978] reported by Pourang et al.[20]; interrater reliability calculated 6 times: ICC=[0.645-945] reported by Kumar et al.[14]) with a high quality of evidence for interrater reliability and a moderate quality of evidence for intrarater reliability. This moderate quality of evidence of intrarater reliability is due to lack of information regarding whether the VASI score was based on a live assessment or a photograph on both occasions in the study of Komen et al.[13]. Moreover, Rosmarin et al.[23] employed a considerable time interval between the two assessments (12 to 24 weeks), without presenting evidence

of the disease's effective stability. Instead they relied on patients' self-assessment using the PaGIC-V score, which is regarded as an unvalidated instrument. Furthermore, Pourang et al.[20] utilized a 7-day interval, which may be considered insufficient to exclude recall bias. A minimum of 2 weeks is recommended. Additionally, interrater reliability is assessed for a modified version of VASI, where response options are limited to increments of 10% (e.g., 10%-20%, etc.) (calculated 3 times: ICC = 0.957, 0.443 and 0.721 respectively)[20]. This resulted in an insufficient rating (due to <70% of the ICC's are >0.7), with a moderate quality of evidence. The downgrade to moderate quality of evidence stems from reliance on only one article for the summarized rating.

The smallest detectable change (SDC) was determined to be 7.1% for interrater reliability and 4.7% for intrarater reliability[13]. However, the **measurement error** was rated as indeterminate because the Minimal Important Difference (MID) had not been calculated within a similar study population.

2. Quality assessment of Measurement Properties of VES (Vitiligo Extent Score)

For VES, no content validity studies were reported. Cronbach's alpha was calculated between BSA-hand units and VES[16]. However, this does not align with internal consistency, hence these results were not taken into account. Reliability was extensively examined in four different studies. **Interrater reliability** received a sufficient rating with a high quality of evidence (2 analyses: ICC=0.923, 0.924 respectively, reported by van Geel et al.[32]; 5 analyses: ICC=[0.79-0.91] reported by van Geel et al.[28]; ICC=0.95 reported by Mehri et al.[15]; ICC=0.997 reported by Chaweekulrat et al.[6]). **Intrarater reliability** received a sufficient rating with a high quality of evidence (2 analyses: ICC= 0.922, 0.943 respectively, reported by van Geel

et al.[32]; 10 analyses: ICC=[0.85-0.98] reported by van Geel et al.[28]; 1 analysis: ICC=0.96 reported by Mehri et al.[15]) . To evaluate the value of using pictures specifically, in addition, the reliability between photos and live assessment was investigated for VES (ICC=0.922 reported by van Geel et al.[32]).

The SDC was calculated eight times by van Geel et al.[32] (SDC=[3.81-11.63%]) and once by Chaweekulrat et al.[6] (SDC=2.71%). Moreover, Uitentuis et al.[27] reported the term 'MID' (=0.2%) for VES, but is not included in this review. The reason is that the 0.2% value actually represents the SSD and not MID. This is because the anchor question assessed only the change in extent and not the smallest change in disease extent that is important for the patient, which is essential to determine the MID[9, 24]. Moreover, the value, considered as MID in the article, was compared to a SDC value from a different study (different population). Due to insufficient information on patient characteristics, it is unclear if these study populations are similar enough. To conclude, the MID within similar study populations was not calculated, making it impossible to rate **measurement error**.

Convergent construct validity received a sufficient rating with a high quality of evidence. This was because the correlation with a validated instrument featuring a similar construct was sufficiently high (VES vs. VASI r=0.909 reported by Mogawer et al.[16]; 3 analyses showing VES vs. VASI: r>0.9 reported by van Geel et al.[32]; VES vs. VASI: r=0.976 and VES vs. SA-VES: r=0.890 reported by Chaweekulrat et al.[6]).

Finally, **responsiveness** was investigated by van Geel et al.[28] and received a sufficient rating with a moderate quality of evidence. This was because the results aligned with the authors' predetermined hypotheses and a logical rationale; 1) the percentage repigmentation after ultraviolet B would be ≥ 10% higher (on average)

compared with topical treatments; 2) in patients with improvement, lesions on the face would have ≥10% higher repigmentation than lesions on the hands; and 3) there would be a coefficient of rank correlation ≥0.5 between the method and global assessment score. The third hypothesis, comparing VES to a 'global assessment score,' was not rated because the exact nature of the score couldn't be ascertained, and thus it was considered as a non-validated instrument being compared. Since the other two hypotheses were deemed valid by the authors (exact results not provided), this did not impact the responsiveness rating.

3. Quality assessment of Measurement Properties of VESplus (Vitiligo Extent Score-plus)

VESplus is a modified version of VES that takes into account repigmentation. No content validity studies were conducted for VESplus, however **interrater reliability** (5 analyses, all ICC= [0.87-0.91] reported by van Geel et al.[28]; 5 analyses: ICC=[0.85-0.97] reported by van Geel et al.[36]) and **intrarater reliability** (10 analyses: ICC=[0.80-0.98] reported by van Geel et al.[28]; 2 analyses: ICC [0.94-0.98] reported by van Geel et al.[36]) were rated as sufficient with a high quality of evidence.

The SDC was calculated (5 analyses [0.5-1.11% BSA] reported by van Geel et al.[36]), but MID for VESplus had not yet been calculated, so no rating could be given for **measurement error**.

For **convergent construct validity**, the summarized rating was sufficient with a high quality of evidence, which is based on following results. The correlation was sufficiently high with VES (r= 0.96, 0.98 respectively reported by van Geel et al.[36]) and VASI (r=0.936 (before UVB), r=0.97 (after UVB treatment course) reported by Youssef et al.[38]) (2 analyses (r= 0.96, 0.98 respectively) reported by van Geel et al.[36].

Finally, for **responsiveness**, the summarized rating was sufficient with a high quality of evidence, which is based on following results. van Geel et al.[28] received a sufficient rating due to the alignment of the results with the authors' predefined hypotheses, which are the same as those used to evaluate the responsiveness of VES, as discussed in the VES section. However, it's worth noting that like VES, the specific results of this assessment were not provided in the article by van Geel et al.[28]. Youssef et al.[38] reported a correlation between VASI and VESplus of r=0.723 of the change before and after a UVB-treatment course. This aligns with a logical rationale that VESplus-scores will decrease in proportion to VASI scores.

4. Quality assessment of Measurement Properties of F-VASI (Facial Vitiligo Area Scoring Index)

F-VASI is a variation of VASI that uses fingertips instead of palms to estimate the affected area on the face.

No content validity studies were conducted for F-VASI. However, both inter- and intrarater reliability were assessed. **Interrater reliability** received a sufficient/insufficient (+/-) summarized rating with a moderate quality of evidence (due to inconsistency) based on following results. Bae et al.[3] reported 2 ICC's=0.857 (+), 0.903 (+) with a 2 week interval respectively. Mehri et al.[15] reported ICC=0.76 (+), Pourang et al., 2023 reported 2 ICC's= 0.542 (-), 0.811 (+) respectively, Banerjee et al.[4] reported ICC=0.699(-). For **intrarater reliability** F-VASI received a sufficient rating with a high quality of evidence, based on the following results. Bae et al.[3] reported ICC=0.903, Mehri et al.[15] reported ICC=0.77, Rosmarin et al.[23] reported 2 ICC's= 0.97, 0.95 respectively, Banerjee et al.[4] reported ICC=0.979, Pourang et al.[20] reported 12 ICC's= [0.175-0.991].

Measurement error could not be rated because the MID was not calculated within a similar study population, which is necessary according to Speeckaert et al.[24]. The SDC was 2.2 fingertip units.

Regarding **convergent construct validity**, no rating could be assigned due to the correlation being calculated with a non-validated instruments (digital image analysis system in Bae et al.[3] and 'Physician's Global Vitiligo Assessment' in Rosmarin et al.[23]). Moreover, Rosmarin et al. did not report correlation results.

Discriminative construct validity was mentioned in the study by Rosmarin et al.[23], wherein the study population was categorized into mild, moderate, and severe disease groups based on their 'Physician's Global Vitiligo Assessment' scores. Subsequently, p-values were calculated, revealing a significant difference in F-VASI scores among the groups. However, no correlation was reported, which is essential for making an assertion regarding construct validity. Subsequently, an 'indeterminate' rating was given for this MP.

Responsiveness in F-VASI was measured by Rosmarin et al.[23]. This after 24 weeks, where the correlation with the 'Patient Global Impression of Change–Vitiligo' (PaGIC-V) scores was calculated (correlation coefficient=0.46). PaGIC-V is considered to be an unvalidated instrument, rendering the quality of evidence very low. The authors did not propose a hypothesis. However, this correlation aligns with the generic hypothesis suggested in the COSMIN guideline, which presumes that the correlation between related but dissimilar constructs should fall between 0.3 and 0.5. Therefore, responsiveness was rated sufficient.

5. Quality assessment of Measurement Properties of VESTA (Vitiligo Extent Score for a Target Area)

No content validity studies were conducted for VESTA, however it received a sufficient rating for both **intrarater reliability** (ICC=0.944) and **interrater reliability** (based on 2 analyses, both with ICC > 0.9) as reported by Bae et al.[2]. However, these ratings come with a moderate quality of evidence.

The smallest detectable change was calculated by Bae et al.[2] (3 analyses: [5.9-9.5%]). However, the MID for VESTA has not yet been determined, therefore a rating for **measurement error** cannot be provided.

Convergent construct validity was examined by comparing the VESTA score with an image analysis system, but no validation studies were found for this comparative instrument. As a result, a rating for convergent construct validity couldn't be assigned, and the quality of evidence for this measurement property is considered very low.

6. Quality assessment of Measurement Properties of PGA extent (Physician Global assessment for extent)

No content validity studies were conducted for PGA-extent. **Interrater reliability** was assessed by raters from different continents, however it received an insufficient rating for **interrater reliability** (ICC=0.67[35]) because ICC should be ≥ 0.7 according to the COSMIN guidelines. This rating received a moderate quality of evidence.

However, **convergent construct validity** was deemed sufficient because of the high correlations observed with instruments featuring a similar construct (PGA-extent vs. VES: r = 0.932; PGA-extent vs. SA-VES: r = 0.877[35]). The correlation between PGA-extent and the BSA 1% rule (considered as VASI) was also calculated, resulting in a

correlation of PGA-extent vs. BSA r = 0.932[35]. This rating received a moderate quality of evidence.

7. Quality assessment of Measurement Properties of PRI (Potential Repigmentation Index)

Responsiveness over a 6-month period of UVB therapy was examined for PRI[5], but could not be assessed. This limitation arises from two key factors. Firstly, the comparison was made with a non-validated instrument, namely the global repigmentation rate. Secondly, subgroup analyses were conducted, employing the t-paired test as the statistical method. It's important to note that the COSMIN guidelines highlight that a paired-t-test is considered an inappropriate measure of responsiveness. This is because it assesses significant change rather than valid change and is highly dependent on the sample size of the study.

8. Quality assessment of Measurement Properties of VDIS 15 & 60 (Vitiligo Disease Improvement Score) and VDAS 15 & 60 (Vitiligo Disease Activity Score)

In this section, four distinct instruments are being discussed, wherein VDIS 15 and VDAS 15 serve as concise versions of their counterparts, VDIS 60 and VDAS 60, respectively[34].

In the context of **content validity**, VDIS and VDAS underwent pilot testing twice consecutively, involving 7 and 5 raters[34]. Some adjustments were made based on the pilot sessions; however, specific details regarding this process were not provided. The article provides a clear explanation of the concept elicitation process. In according to the COSMIN guidelines, concept elicitation can be considered as relevance. However, the assessment of relevance could not be rated, as it remains uncertain whether the

examination of the appropriateness of response options and the recall period was conducted.

All four instruments underwent examination for **interrater reliability**, resulting in the following scores: VDAS15: ICC=0.87; VDIS15: ICC=0.78; VDAS60: ICC=0.91; VDIS60: ICC=0.80[34]. **Intrarater reliability** was also evaluated, yielding these scores: VDAS15: ICC=0.865; VDIS15: ICC=0.781; VDAS60: ICC=0.913; VDIS60: ICC=0.800[34]. In all cases, they were rated as sufficient with a moderate quality of evidence.

For **convergent construct validity**, these instruments were compared to VESplus, which has a related but unsimilar construct. In this case, the correlations were sufficiently high (r > 0.3): VDAS15: r=0.79; VDIS15: r=0.372; VDAS60: r=0.795; VDIS60: r=0.486[34]. This rating received a high quality of evidence. VDIS and VDAS were also compared to the 'PGA expert global disease progression score.' However, no sources or validation studies for this score were located, leading to considering it as a non-validated instrument, and consequently, no rating could be provided.

Discriminative construct validity was deemed sufficient for all four instruments. This conclusion was reached because the logical rationale established by van Geel et al.[34] was satisfied for both VDAS15 and VDAS60: "We expect that patients with more than 5 involved body locations will have at least a 10% higher VDAS compared to patients with less than 6 involved body locations." For VDAS 15, this difference was 291% higher, and for VDAS 60, it was 303% higher. Regarding VDIS15 and VDIS 60, the authors formulated the following logical hypothesis: "The mean repigmentation score on the face will be ≥20% higher compared to that of the hands." This hypothesis held

true, with VDIS15 being 69% higher and VDIS60 being 93% higher. The rating for discriminative construct validity received a moderate quality of evidence.

9. Quality assessment of Measurement Properties of VSAS (Vitiligo Signs of Activity Score)

Regarding **content validity**, relevance and comprehensiveness were rated as 'indeterminate as it was not mentioned in the paper of van Geel et al.[33] that a qualitative method (open-ended questions) was used to determine comprehensiveness or relevance of several proposed activity items (survey to vitiligo experts) so it remains unclear if data saturation was reached. Additionally, in this study, the survey was being administered to fewer than 30 vitiligo experts (which is the lower limit for reliably gathering data using a quantitative method such as a closed (multiple-choice) question survey).

Furthermore, VSAS achieved a sufficient rating for **interrater reliability** (2 analyses: ICC=[0.87-0.9][33]) and **intrarater reliability** (3 analyses: ICC=[0.86-0.95][33]). Both ratings received a moderate quality of evidence.

For **convergent construct validity**, a sufficient summarized rating was given with a high quality of evidence. This conclusion was based on a positive rating based on the results of van Geel et al.[33]. This rating corresponds to the fulfillment of three out of the four hypotheses (75%) articulated by the authors: The first hypothesis, which aimed for "A positive correlation of ≥0.5 between VSAS and the global disease activity scores," was successfully met with a median correlation coefficient of 0.75. The second hypothesis posited that "In patients with a median VSAS of 0 to 3, the answer to the PGA overall score would be 'not present' or 'mild' in ≥ 50% of cases compared to patients in other categories." This hypothesis was also fulfilled, as patients with

VSAS scores in the [0-3] range predominantly scored 'not present' or 'mild' in 77.8% of cases. However, the third hypothesis, which anticipated that "In patients with a median VSAS score of 0, the PGA overall score would be 'not present' in ≥30% of cases compared to patients in other categories," which was not met, because only twice the PGA score of 'not present' was selected. This discrepancy could be attributed to the limited number of cases with a VSAS score of 0 (with a mean/median number of cases at 5.5/6) and the fact that the expert rater was not provided with the predefined definitions for instrument use. The final hypothesis, "In patients with a median VSAS score of ≥10, the answer to the PGA overall score would often (in at least 50% of cases) be in the 'severe-very severe' category compared to patients in other categories," was successfully met, with 75% of cases in this subgroup scoring as 'severe' or 'very severe'. An indeterminate rating was assigned to the findings from Youssef et al.[38]. This study examined the correlation between VSAS and the Vitiligo Disease Activity (VIDA) Scale, a PROM assessing the last time point of disease activity. However, three VIDA-validation papers[18, 7, 38] were analyzed, with 2 out of the 3 analyzed MPs rated as negative, indicating no evidence that this is a valid instrument to use as a comparative tool. Hence, the results cannot be interpreted (r = 0.846 after UVB treatment course, r = -0.007 before UVB treatment course[38]). The authors attributed this difference to patients paying more attention to the progression of their disease after treatment compared to before treatment initiation. Additionally, correlations between the subscales of VSAS and VIDA were reported but could also not be intrepreted (VSASKoebner vs. VIDA, both post-UVB treatment course: r = 0.427, VSASKoebner vs. VIDA, both pre-UVB treatment course: r = -0.132, VSASconfetti vs. VIDA, both post-UVB treatment course: r = 0.211, VSASconfetti vs. VIDA, both pre-UVB treatment course: r = -0.015, VSAShypochromic, both post-UVB

treatment course: r = 0.873, VSAShypochromic, both pre-UVB treatment course: r = -0.106)[38] .

10. Quality assessment of Measurement Properties of K-VSCOR (Koebner's phenomenon in vitiligo score)

Regarding **content validity**, specifically **comprehensiveness**, an attempt was made to map all Koebner-sensitive zones based on clinical examinations of 351 patients by two dermatologists (it wasn't specified whether both dermatologists examined all patients)[10]. Given the large number of patients, it can be assumed that saturation was reached, and all locations were mapped, resulting in a sufficient rating. Nevertheless, this rating is supported by a low quality of evidence since it relies solely on data from the instrument's development phase, and crucial information about the methodology was missing in the article.

Diallo et al.'s article[10] focused on the assessment of both **discriminative structural validity** (referred to as 'internal validation' in the article) and **structural validity** (referred to as 'calibration' in the article). However, these measurement properties were not analyzed according to the recommended COSMIN guidelines. Consequently, the results couldn't be interpreted, leading to an 'indeterminate' rating for these MPs. The study also mentioned 'discrimination,' involving AUC-ROC calculations. However, due to the lack of clarity, **responsiveness** could not be rated.

11. Quality assessment of Measurement Properties of VETFa (Vitiligo European Task Force assessment)

VETFa comprises three subscales: depigmentation, staging, and spreading.

In the context of **content validity**; VETFa (all subscales) was developed based on tests in eight different European countries by vitiligo experts within their own patient populations[25]. Additionally, several group discussions were conducted with the aim of summarizing a maximum of relevant disease- and patient-related information[25]. However, it's important to note that the results of these tests and discussions remain unknown. Consequently, comprehensiveness and relevance received an 'indeterminate' rating. This with a low quality of evidence, primarily because critical methodological information was missing.

VETFa-extent received a sufficient rating with a moderate quality of evidence concerning **responsiveness**, based on the analyses conducted by Komen et al.[13], where the following hypotheses were met; the average repigmentation after 6.3 months of UVB treatment measured with VETFa was 30% (hypothesis: >15%). Repigmentation on the face was 42%, while the hands showed only 28% repigmentation during the same UVB treatment period, in line with the hypothesis that the face would show ≥ 10% more repigmentation compared to the hands. Correlations with non-validated instruments, such as 'the percentage of repigmentation scored by a physician' and 'the global assessment of improvement' scored by the patient and the physician, were also examined, but the results were not rated due to lack of validation of these instruments. The sufficient rating for responsiveness received a moderate quality of evidence. It was rated in 33 patients with one rater involved, aligning with the precision criteria, which suggest that either 15 patients and 3 raters or 20 patients and 2 raters are sufficient.

Interrater reliability was rated as sufficient with a moderate quality of evidence for VETFa-extent (ICC=0.88, reported by Komen et al.[13], K-value for VETFa extent not

reported by Taïeb et al.[25]). Concerning VETFa-staging and VETFa-spreading, interrater reliability was rated insufficient with a high quality of evidence for both subscales (VETFa staging: ICC=0.35; VETFa spreading: ICC=0.42 reported by Komen et al.[13]) (VETFa staging: K=0.499; VETFa spreading: K=0.388 reported by Taïeb et al.[25]) However, **intrarater reliability** was rated as sufficient with a moderate quality of evidence for both VETFa-extent and VETFa-staging, but score insufficient with a moderate quality of evidence for VETFa-spreading (VETFa-extent: ICC=0.97; VETFa-staging: ICC=0.84; VETFa-spreading: ICC=0.47 reported by Komen et al.[13]).

The SDC for VETFa-extent was calculated by Komen et al.[13] (for interrater reliability: SDC=10.4%; for intrarater reliability: 2.9%). **Measurement error** could not be rated because the MID was not known within a similar study population.
